# The NuRD Chromatin-Remodeling Enzyme CHD4 Promotes Embryonic Vascular Integrity by Transcriptionally Regulating Extracellular Matrix Proteolysis

**DOI:** 10.1371/journal.pgen.1004031

**Published:** 2013-12-12

**Authors:** Kyle G. Ingram, Carol D. Curtis, Robert Silasi-Mansat, Florea Lupu, Courtney T. Griffin

**Affiliations:** 1 Cardiovascular Biology Research Program; Oklahoma Medical Research Foundation; Oklahoma City, Oklahoma, United States of America; 2 Department of Cell Biology; University of Oklahoma Health Sciences Center; Oklahoma City, Oklahoma, United States of America; 3 Department of Pathology; University of Oklahoma Health Sciences Center; Oklahoma City, Oklahoma, United States of America; Gladstone Institute of Cardiovascular Disease, United States of America

## Abstract

The extracellular matrix (ECM) supports vascular integrity during embryonic development. Proteolytic degradation of ECM components is required for angiogenesis, but excessive ECM proteolysis causes blood vessel fragility and hemorrhage. Little is understood about how ECM proteolysis is transcriptionally regulated during embryonic vascular development. We now show that the NuRD ATP-dependent chromatin-remodeling complex promotes vascular integrity by preventing excessive ECM proteolysis *in vivo*. Mice lacking endothelial CHD4—a catalytic subunit of NuRD complexes—died at midgestation from vascular rupture. ECM components surrounding rupture-prone vessels in *Chd4* mutants were significantly downregulated prior to embryonic lethality. Using qPCR arrays, we found two critical mediators of ECM stability misregulated in mutant endothelial cells: the urokinase-type plasminogen activator receptor (uPAR or *Plaur*) was upregulated, and thrombospondin-1 (*Thbs1*) was downregulated. Chromatin immunoprecipitation assays showed that CHD4-containing NuRD complexes directly bound the promoters of these genes in endothelial cells. uPAR and THBS1 respectively promote and inhibit activation of the potent ECM protease plasmin, and we detected increased plasmin activity around rupture-prone vessels in *Chd4* mutants. We rescued ECM components and vascular rupture in *Chd4* mutants by genetically reducing urokinase (uPA or *Plau*), which cooperates with uPAR to activate plasmin. Our findings provide a novel mechanism by which a chromatin-remodeling enzyme regulates ECM stability to maintain vascular integrity during embryonic development.

## Author Summary

Blood vessels are surrounded by an extracellular matrix (ECM), which provides structural support and helps vessels withstand the biomechanical pressure of flowing blood. As blood vessels grow during embryonic development, the ECM is locally degraded to allow new vessels to sprout from existing ones. This localized ECM degradation must be tightly regulated, however, because excessive matrix breakdown leaves vessels susceptible to rupture and lethal hemorrhage. We now present a novel mechanism by which production of proteins that promote or repress ECM degradation is coordinated. We show that a chromatin-remodeling enzyme called CHD4 is critical for maintaining blood vessel integrity during embryonic development. CHD4 achieves this task by limiting production of proteins that degrade ECM and promoting production of proteins that protect ECM around developing blood vessels. A better understanding of how ECM degradation is regulated during development could have an impact on our ability to combat adult pathologies, such as aneurysms, which are associated with excessive ECM degradation and vascular fragility.

## Introduction

The extracellular matrix (ECM) plays a critical role in maintaining blood vessel integrity by serving as a scaffold to which endothelial cells and vascular support cells can adhere [1], [2]. Signals between ECM components and endothelial cells likewise promote vessel stabilization by influencing endothelial cell proliferation, morphology, and survival. Aberrant degradation of ECM components is associated with life-threatening aortic and cerebral aneurysms, which are characterized by vascular fragility or rupture [3], [4].

ECM composition and stability also impact vascular integrity during embryonic development. Mice with mutations in ECM components or in regulators of ECM deposition die during midgestation with vascular rupture and hemorrhage [5], [6]. The timing of these lethal phenotypes is likely influenced by the combination of weakened vascular ECM and increasing intravascular pressure that results from embryonic heart rate acceleration during midgestation [7]. Mice with mutations that cause excessive vascular ECM degradation also succumb to vascular rupture at midgestation [8], [9]. ECM proteases are produced by activated endothelial cells during vascular development and are necessary for sprouting angiogenesis [10]. However, ECM proteases must be tightly regulated to prevent excessive proteolysis of the vascular matrix and blood vessel fragility. Currently, the transcriptional regulation and coordination of ECM proteolysis during vascular development is poorly understood.

Epigenetic factors are important transcriptional regulators of developmental processes, and increasing evidence supports their roles in cardiovascular development. Enzymes that add or subtract covalent modifications from histone tails affect transcription of target genes that impact heart and blood vessel development [11], [12]. Likewise, ATP-dependent chromatin-remodeling complexes, which transiently displace nucleosomes at gene regulatory regions, mediate transcriptional regulation of genes involved in cardiovascular development [13], [14]. Mammalian Nucleosome-Remodeling and Histone Deacetylase (NuRD) chromatin-remodeling complexes contain both histone-modifying and chromatin-remodeling enzymes [15]–[17]. These multi-subunit complexes incorporate histone deacetylases 1 and 2 (HDAC1 and HDAC2), which remove acetyl groups from histone tails. NuRD complexes also contain ATPase catalytic subunits belonging to the SNF2 superfamily: the Chromodomain-Helicase-DNA-binding proteins CHD3 and CHD4 (also called Mi-2α and Mi-2β, respectively). CHD3 and CHD4 contain protein- and DNA-binding domains in addition to an ATPase domain that provides energy for nucleosome remodeling [18], [19]. NuRD complexes were traditionally thought to act in a transcriptionally repressive manner, since histone deacetylation is associated with gene silencing. However, evidence from developing blood and nerve cell lineages indicates that NuRD complexes can facilitate target gene activation in addition to silencing [20]–[22]. Although our lab previously showed NuRD negatively regulates vascular Wnt signaling in the developing extraembryonic yolk sac [23], a role for NuRD in the embryonic vasculature has not yet been described.

We now report that embryos depleted of the NuRD ATPase CHD4 in vascular endothelial cells died by embryonic day 11.5 (E11.5) with blood vessel rupture and massive hemorrhage. Our studies reveal coordinated chromatin-based mechanisms by which CHD4-containing NuRD complexes transcriptionally regulate ECM proteolysis to maintain vascular integrity during embryonic development.

## Results

### *Chd4^fl/fl^;Tie2-Cre^+^* Embryos Die at Midgestation

**Figure 1:**
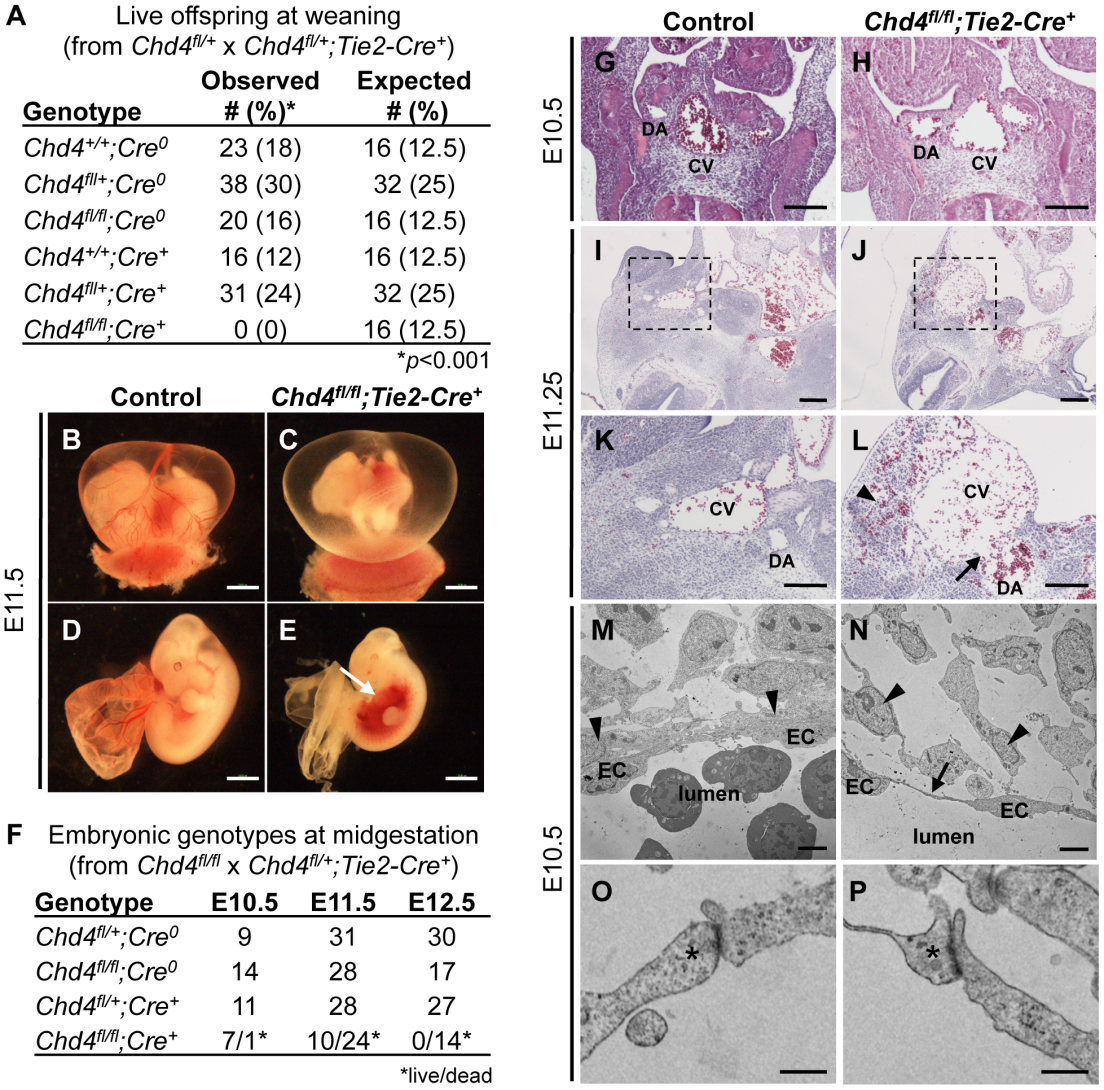
*Chd4^fl/fl^;Tie2-Cre^+^* embryos undergo vascular rupture by E11.25. (A) *Chd4^fl/+^* females were mated with *Chd4^fl/+^;Tie2-Cre^+^* males, and live progeny from 22 litters were genotyped and scored at weaning. No live *Chd4^fl/fl^;Tie2-Cre^+^* mice were recovered [χ^2^(5_dof_): *p*<0.001]. (B–E) Gross images of E11.5 littermate control (B,D) and *Chd4^fl/fl^;Tie2-Cre^+^* (C,E) embryos. Arrow in panel E indicates massive hemorrhage within the ventral trunk region of a *Chd4^fl/fl^;Tie2-Cre^+^* embryo. (F) *Chd4^fl/fl^* females were mated with *Chd4^fl/+^;Tie2-Cre^+^* males, and dissections were performed on E10.5–12.5 embryos. Dead embryos were characterized by absence of heartbeat and onset of necrosis. No surviving *Chd4^fl/fl^;Tie2-Cre^+^* embryos were found at E12.5 [χ^2^(3_dof_): *p*<0.01]. (G–L) Hematoxylin and eosin (H&E) staining of E10.5 (G,H) or E11.2±5 (I–L) littermate control (G,I,K) and *Chd4^fl/fl^;Tie2-Cre^+^* (H,J,L) embryos. Boxed regions in panels I and J are shown at higher magnification in panels K and L respectively. Arrow in panel L reveals site of vascular rupture between the mutant dorsal aorta and cardinal vein; arrowhead indicates blood in extravascular tissues. DA, dorsal aorta; CV, cardinal vein. (M–P) Transmission electron micrographs from vessel walls of E10.5 littermate control (M,O) and *Chd4^fl/fl^;Tie2-Cre^+^* (N,P) embryos. Arrowheads in panels M and N indicate smooth muscle cells adjacent to endothelial cells (ECs). Arrow in panel N points to a long, thin EC extension. (O,P) Endothelial cell junctions (*) are intact in both control and mutant sections. Scale bars: 1 mm (B–E); 100 µm (G–L); 2 µm (M,N); 500 nm (O,P).

We previously deleted *Chd4* from embryonic endothelial cells with a *Tie2-Cre* transgenic mouse line in order to assess the role of CHD4-containing NuRD complexes in vascular development. We found Wnt signaling was upregulated in *Chd4^fl/fl^;Tie2-Cre^+^* yolk sac vessels at E10.5, but embryonic vascular phenotypes were not apparent at this developmental time point [23]. In order to determine whether *Chd4^fl/fl^;Tie2-Cre^+^* embryos could survive development, we mated *Chd4^fl/+^* and *Chd4^fl/+^;Tie2-Cre^+^* mice together and expected to obtain 12.5% *Chd4^fl/fl^;Tie2-Cre^+^* offspring. However, no *Chd4^fl/fl^;Tie2-Cre^+^* animals were detected at weaning (Figure 1A). These results indicated that expression of *Chd4* on developing endothelial cells was important for embryonic survival.

Since many mutants with vascular defects die during midgestation [24], we focused on this time period for preliminary analysis of our *Chd4^fl/fl^;Tie2-Cre^+^* embryos. CHD4 is broadly expressed at E10.5, and we detected it by immunostaining in endothelial cells of both large and small vessels (Figure S1). We performed dissections on E10.5–12.5 embryos and consistently found *Chd4^fl/fl^;Tie2-Cre^+^* embryos with massive hemorrhage in the trunk region at E11.5 (Figure 1E). *Chd4^fl/fl^;Tie2-Cre^+^* embryos and yolk sacs appeared pale in comparison to littermate controls due to pooling of embryonic blood (Figure 1B–1E). Prior to hemorrhage, mutant embryos were visibly normal and displayed minimal developmental delay compared to E10.5 littermate controls. Thus, we determined that *Chd4^fl/fl^;Tie2-Cre^+^* embryos died at E11.0–11.75 from sudden and massive hemorrhage, since embryos appeared normal at E10.5 and were found dead by E12.5 (Figure 1F).

### *Chd4^fl/fl^;Tie2-Cre^+^* Dorsal Aortae and Cardinal Veins Are Prone to Rupture

The localized hemorrhage observed at E11.5 suggested vascular rupture in the trunk region of *Chd4^fl/fl^;Tie2-Cre^+^* embryos. Histological analysis revealed rupture of the dorsal aortae and cardinal veins of *Chd4^fl/fl^;Tie2-Cre^+^* embryos at E11.25 (Figure 1J and 1L), corresponding closely with the time of death. However, there was no indication of impending vascular rupture or evidence of blood leakage from *Chd4^fl/fl^;Tie2-Cre^+^* dorsal aortae or cardinal veins at E10.5 (Figure 1H). Vascular patterning within *Chd4^fl/fl^;Tie2-Cre^+^* embryos was largely normal at E10.5 (Figure S2A–S2F). Likewise, vascular patterning was comparable in control and *Chd4^fl/fl^;Tie2-Cre^+^* yolk sacs at E10.5 (Figure S2G–S2K), as we previously reported [23]. *Chd4^fl/fl^;Tie2-Cre^+^* hearts showed slight evidence of hypotrabeculation compared to littermate controls at E10.5 (Figure S3D). This hypotrabeculation was accompanied by a subtle decrease in cardiac ECM, as assessed by Alcian blue staining (Figure S3B). Since cardiac ECM is critical for supporting trabeculation [25], we suspect that *Chd4^fl/fl^;Tie2-Cre^+^* hypotrabeculation is secondary to reduced cardiac ECM. Nevertheless, hypotrabeculation and diminished cardiac ECM are not associated with vascular rupture in other mutants [25]–[30]. Therefore the sudden vascular rupture and lethal hemorrhage in *Chd4^fl/fl^;Tie2-Cre^+^* embryos did not likely result from grossly observable cardiovascular anomalies.

### *Chd4^fl/fl^;Tie2-Cre^+^* Endothelial Cells Have Long Cytoplasmic Extensions but Intact Intercellular Junctions Prior to Vascular Rupture

In order to evaluate the morphology of *Chd4^fl/fl^;Tie2-Cre^+^* endothelial cells prior to vascular rupture, we processed E10.5 control and mutant littermate embryos for transmission electron microscopy (TEM). We found that endothelial cells lining *Chd4^fl/fl^;Tie2-Cre^+^* vessels had long, thin extensions between cells that were not seen in control endothelial cells (Figure 1M and 1N). In addition, we observed smooth muscle cells attached tightly to endothelial cells outside control dorsal aortae (Figure 1M, arrowheads), but these attachments were disrupted outside *Chd4^fl/fl^;Tie2-Cre^+^* dorsal aortae (Figure 1N). These phenotypes indicated that *Chd4^fl/fl^;Tie2-Cre^+^* vessels were fragile and lacked closely apposed supporting smooth muscle cells prior to rupture.

We also evaluated *Chd4^fl/fl^;Tie2-Cre^+^* endothelial cell proliferation and viability prior to vascular rupture. At E10.5, no differences were seen in numbers of proliferating endothelial cells within dorsal aortae of control and *Chd4^fl/fl^;Tie2-Cre^+^* embryos, as assessed by immunostaining with antibodies against the proliferation markers phosphorylated histone H3 (PPH3) or Ki67 (Figure S4A–S4D). Likewise no differences were seen in numbers of apoptotic endothelial cells in control and *Chd4^fl/fl^;Tie2-Cre^+^* embryos following TUNEL staining or immunostaining with an antibody against active Caspase 3 (Figure S4E–S4H). We concluded that the long endothelial cell extensions and loosely connected smooth muscle cells lining *Chd4^fl/fl^;Tie2-Cre^+^* vessels at E10.5 were not influenced by aberrant endothelial cell apoptosis or deficient proliferation.

Since mutant embryos with defective endothelial cell junctions can display thin, extended endothelial cells prior to hemorrhagic events [31], we analyzed endothelial junctional components and morphology in control and *Chd4^fl/fl^;Tie2-Cre^+^* embryos prior to vascular rupture. We confirmed that intercellular junctions appeared morphologically similar by TEM in control and *Chd4^fl/fl^;Tie2-Cre^+^* endothelial cells at E10.5 (Figure 1O and 1P). Likewise, E10.5 control and *Chd4^fl/fl^;Tie2-Cre^+^* dorsal aortae immunostained with antibodies against the adherens junction protein vascular endothelial cadherin (VE-cadherin; Figure S5A and S5B) or the tight junction protein zonula occludens-1 (ZO-1; Figure S5C and S5D) revealed comparable levels and patterns of expression. Therefore, our data suggested that defective cell junctions did not contribute to the thin endothelial cell extensions we saw at E10.5 or to vascular rupture at E11.5.

### Extracellular Matrix Components Are Diminished around *Chd4^fl/fl^;Tie2-Cre^+^* Rupture-Prone Vessels

**Figure 2:**
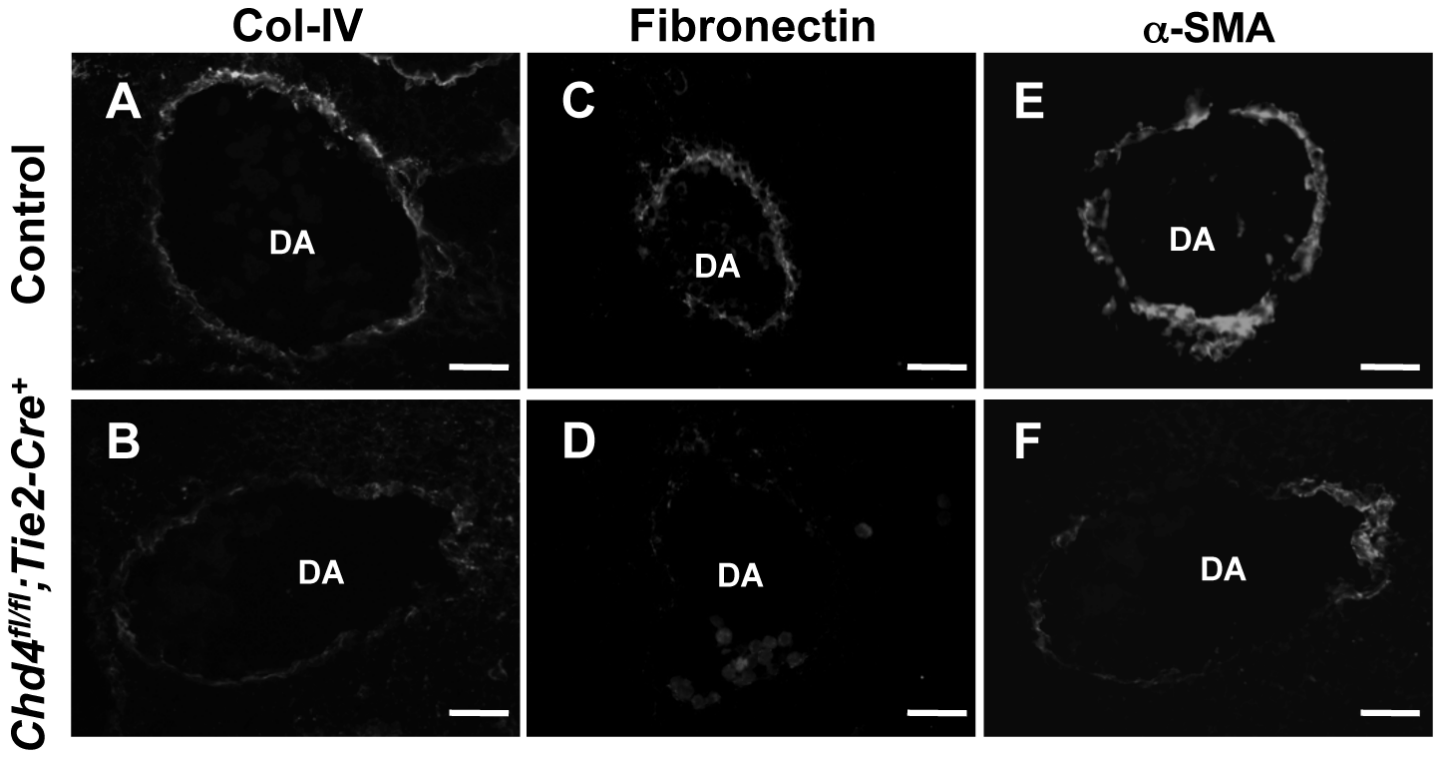
Extracellular matrix (ECM) components are diminished around *Chd4^fl/fl^;Tie2-Cre^+^* vessels prior to rupture. Histological sections of dorsal aortae (DA) from E10.5 littermate control (A,C,E) and *Chd4^fl/fl^;Tie2-Cre^+^* (B,D,F) embryos were stained with antibodies against the ECM basement membrane component type IV collagen (Col-IV; A,B) or the ECM component fibronectin (C,D) or against the smooth muscle cell differentiation marker α-SMA (E,F). Representative images from three independent experiments on separate sets of embryos are shown. Scale bars: 50 µm (A–F).

In addition to endothelial cell junctions, the extracellular matrix plays an important role in the maintenance of vascular integrity [2]. One element of the ECM, the basement membrane, is a sheet-like structure that lines and supports endothelial cells. We immunostained sections of control and *Chd4^fl/fl^;Tie2-Cre^+^* embryos with an antibody against type IV collagen—a major component of the basement membrane—to assess its expression around rupture-prone dorsal aortae. Type IV collagen was diminished around *Chd4^fl/fl^;Tie2-Cre^+^* versus control dorsal aortae at E10.5 (Figure 2A and 2B). Likewise, fibronectin, another ECM component that is abundantly expressed around dorsal aortae at midgestation [32], was downregulated around *Chd4^fl/fl^;Tie2-Cre^+^* versus control dorsal aortae at E10.5 (Figure 2C and 2D). Therefore ECM components were diminished around *Chd4^fl/fl^;Tie2-Cre^+^* dorsal aortae prior to the vascular rupture we consistently saw in mutant embryos at E11.5. Interestingly, type IV collagen and fibronectin were not significantly diminished around small vessels we examined in mutant limb buds (data not shown), and these microvessels were not prone to rupture in *Chd4^fl/fl^;Tie2-Cre^+^* mutants.

Because smooth muscle cells (SMCs) provide mechanical support for developing arteries, we also assessed E10.5 *Chd4^fl/fl^;Tie2-Cre^+^* dorsal aortae for expression of the SMC differentiation marker alpha-smooth muscle actin (α-SMA). Cells surrounding *Chd4^fl/fl^;Tie2-Cre^+^* dorsal aortae expressed less α-SMA than those surrounding control dorsal aortae (Figure 2E and 2F). Since α-SMA expression is influenced by ECM components such as type IV collagen [33], we suspected the reduced type IV collagen around rupture-prone *Chd4^fl/fl^;Tie2-Cre^+^* dorsal aortae contributed to diminished α-SMA levels on SMCs around those vessels. Alternatively, the reduced α-SMA expression and loose attachments between endothelial cells and SMCs observed by TEM (Figure 1N) could indicate that mutant endothelial cells failed to recruit SMCs effectively. To address this possibility, we examined expression of several genes implicated in SMC recruitment toward endothelium in E10.5 control versus *Chd4^fl/fl^;Tie2-Cre^+^* endothelial cells (*Tgfb1*, *Tgfbr1*, *Eng*, *Angpt1*, *Angpt2*, and *S1pr1*; Table S1) or in an endothelial cell line depleted of CHD4 (*Hbegf* and *Tgfbr2*; Figure S6). We did not see significant expression changes in these genes, although we did see downregulation of *Tie2* (*Tek*; Table S2), which plays important roles in communication between endothelial cells and SMCs [34]. Additionally, we saw significant upregulation of *Pdgfb* in CHD4-depleted endothelial cells (Figure S6), which reduces α-SMA expression on vascular SMCs [35], [36]. Therefore, α-SMA expression on cells surrounding rupture-prone *Chd4^fl/fl^;Tie2-Cre^+^* vessels could be diminished due to multiple causes. Overall, we hypothesized that *Chd4^fl/fl^;Tie2-Cre^+^* vessels were susceptible to rupture because their walls were weakened from a paucity of ECM components and differentiated SMCs.

### CHD4 Directly Inhibits Expression of uPAR

Since CHD4 typically exerts its cellular actions through transcriptional regulation of target genes, we sought to determine which endothelial CHD4 target genes could impact ECM composition. We isolated endothelial cells from E10.5 littermate control and *Chd4^fl/fl^;Tie2-Cre^+^* and embryos and identified significant gene expression changes using two commercially available quantitative real-time PCR (qPCR) arrays containing a total of 157 genes. We chose these arrays because they contained many genes with important roles in ECM composition or proteolysis. Notably, we did not see significant downregulation of genes encoding midgestational ECM components, such as collagens, laminins, and fibronectin (Table S1). Nor did we see upregulation of matrix metalloproteinases that could contribute to ECM proteolysis. However, the arrays revealed the urokinase-type plasminogen activator (uPA, encoded by the gene *Plau*) was significantly upregulated in *Chd4^fl/fl^;Tie2-Cre^+^* endothelial cells (Table S2). This result was notable since we had previously identified the uPA receptor (uPAR, encoded by the gene *Plaur*) as a CHD4 target gene in yolk sac vessels but had not determined the functional relevance of its upregulation in *Chd4^fl/fl^;Tie2-Cre^+^* endothelial cells [23]. uPAR regulates ECM proteolysis by binding and localizing uPA to cell surfaces where it activates the serine protease plasmin [37]. Therefore, we hypothesized that upregulation of uPA/uPAR and subsequent plasmin activity might contribute to ECM fragility, vascular rupture, and lethality in *Chd4^fl/fl^;Tie2-Cre^+^* embryos.

**Figure 3:**
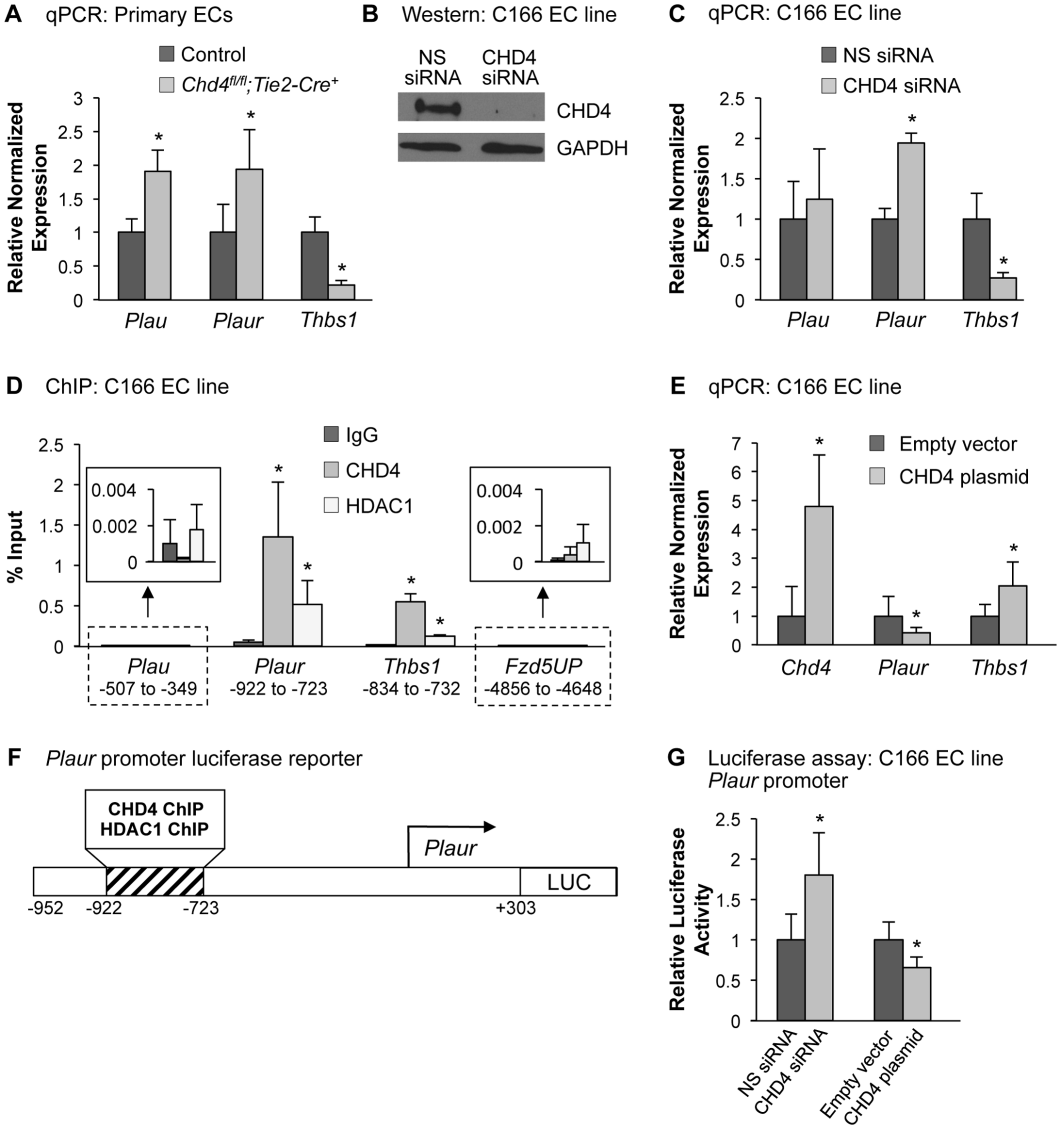
CHD4 differentially regulates *Plaur* and *Thbs1* expression in endothelial cells. (A) qPCR with gene-specific primers for *Plau*, *Plaur*, and *Thbs1* was performed on endothelial cells isolated from E10.5 littermate control and *Chd4^fl/fl^;Tie2-Cre^+^* embryos. Data were normalized to the relative expression of control samples. Error bars represent SD of results from three independent experiments. (B and C) C166 endothelial cells were transfected with nonspecific (NS) or CHD4-specific siRNA oligonucleotides for 24 h. (B) Western blot analysis was performed on cell lysates using antibodies that recognize CHD4 or GAPDH. A representative blot from 3 independent experiments is shown. (C) RNA was isolated, cDNA was synthesized, and qPCR was performed using *Plau*-, *Plaur*-, or *Thbs1*-specific primers. Data were normalized to the relative expression of NS siRNA-treated samples. Error bars represent SD of results from three to four independent experiments. (D) Chromatin immunoprecipitation (ChIP) assays were carried out in C166 endothelial cells using antibodies against normal mouse IgG (negative control), CHD4, or HDAC1. Immunoprecipitated DNA was analyzed by qPCR to examine CHD4 and HDAC1 enrichment at the *Plau*, *Plaur*, and *Thbs1* promoters. A transcriptionally inactive region approximately 5 kb upstream of the *Fzd5* transcription start site (*Fzd5UP*) was assessed as a negative control for CHD4 and HDAC1 binding. Data are represented as a percent of total input chromatin. Error bars represent SD of results from three independent experiments. Results for *Plau* and *Fzd5UP* ChIP experiments are magnified in the insets. For the *Plaur* and *Thbs1* ChIPs, CHD4 and HDAC1 binding were statistically compared against IgG binding at the respective loci or against CHD4 and HDAC1 binding at the *Fzd5UP* locus; both sets of comparisons revealed significant enrichment. (E) qPCR with gene-specific primers for *Chd4*, *Plaur*, and *Thbs1* was performed on C166 endothelial cells transfected with 0.02 ng of a CHD4 expression plasmid or the analogous empty vector backbone (control). Data were normalized to the relative expression of control samples. Error bars represent SD of results from three independent experiments. (F) Schematic of the region of the murine *Plaur* promoter that was cloned into a luciferase (LUC) reporter plasmid for use in G. The *Plaur* promoter fragment encompasses the region to which CHD4 and HDAC1 were shown to bind by ChIP in D. (G) Luciferase assays were performed in C166 cells co-transfected with 250 ng of the reporter schematized in F and 10 ng of a constitutive Renilla luciferase control plasmid. Cells were also transfected with either 10 pmol of non-specific (NS) siRNA or CHD4 siRNA oligonucleotides to knock down endogenous CHD4 or with the CHD4 expression plasmid or its relevant control (empty vector) described in E. All transfections were performed for 24 h. Ratios of relative luciferase∶renilla activity were normalized to results from the control samples. Error bars represent SD of results from four independent experiments (with triplicate samples) for the siRNA-transfected samples and from five independent experiments (with triplicate samples) for the CHD4/control plasmid-transfected samples. All statistical calculations for Figure 3 were performed using a two-tailed Student's *t* test (*, *p*<0.05).

In order to validate the qPCR array results and determine whether *Plau* expression was altered in our mutant embryos prior to vascular rupture, we isolated endothelial cells from E10.5 control and *Chd4^fl/fl^;Tie2-Cre^+^* embryos and performed qPCR using primers specific for *Plau* and *Plaur*. Indeed, we found both *Plau* and *Plaur* were significantly upregulated in *Chd4^fl/fl^;Tie2-Cre^+^* endothelial cells (Figure 3A). We next performed chromatin immunoprecipitation (ChIP) assays to determine whether *Plau*—like *Plaur*—is a direct CHD4 target gene. In order to obtain the number of cells required for ChIP, we used the C166 yolk sac-derived murine endothelial cell line [38]. We chose this cell line for ChIP because knockdown of CHD4 in C166 cells resulted in upregulation of *Plaur* mRNA (Figure 3C), as we saw in primary *Chd4^fl/fl^;Tie2-Cre^+^* endothelial cells. *Plau* expression trended upward as well in CHD4 knockdown C166 cells, but it was not significantly upregulated like *Plaur* (Figure 3C). We found no evidence that CHD4 bound the *Plau* promoter in wildtype C166 cells by ChIP but confirmed that CHD4 bound the *Plaur* promoter in a region approximately 900 bp upstream of the transcription start site (TSS) (Figure 3D). Therefore, our data indicated that CHD4 directly inhibited *Plaur*—but not *Plau*—expression in endothelial cells. Since *Plau* gene expression is influenced by uPAR signaling [39], [40], we suspected that the upregulated *Plau* expression we saw in *Chd4^fl/fl^;Tie2-Cre^+^* endothelial cells was secondary to upregulated *Plaur*. Importantly, we also detected binding of HDAC1 to the same region of the *Plaur* promoter at which we detected CHD4 binding by ChIP (Figure 3D). Since HDAC1 can participate in NuRD complexes [15], these data indicated that CHD4/HDAC1-containing NuRD complexes repressed *Plaur* transcription in endothelial cells.

To further assess the impact of CHD4 on *Plaur* transcription, we transfected a CHD4 expression plasmid into C166 cells and assessed *Plaur* transcription by qPCR. We detected significant downregulation of *Plaur* with the overexpression of CHD4 (Figure 3E), which complemented our genetic and knockdown data and suggested that CHD4 inhibited *Plaur* transcription. Finally, we generated a luciferase reporter containing a fragment of the *Plaur* promoter that included the region in which we detected positive CHD4 and HDAC1 binding by ChIP (Figure 3F), and examined reporter activity in C166 endothelial cells. We saw a significant increase in reporter activity upon knockdown of endogenous CHD4, and overexpression of CHD4 significantly decreased reporter activity (Figure 3G). Altogether, these data indicated that CHD4 directly and negatively regulated *Plaur* transcription in endothelial cells.

To assess how CHD4 mediated negative regulation of *Plaur* transcription, we analyzed the acetylation status of H3K9 and H3K27, two covalent histone marks associated with transcriptional activation that can be influenced by NuRD deacetylase activity [41], [42]. Following CHD4 knockdown we saw no significant changes in H3K9Ac or H3K27Ac marks in the region of the *Plaur* promoter that bound CHD4 and HDAC1 (Figure S7). Therefore CHD4/HDAC1-containing NuRD complexes do not appear to repress *Plaur* transcription through deacetylation of H3K9 or H3K27 in the promoter region we analyzed. Further analysis of the *Plaur* promoter in CHD4 knockdown cells will be required to discern the precise mechanism by which NuRD negatively regulates *Plaur* transcription in endothelium.

### CHD4 Directly Promotes Expression of Thrombospondin-1

In addition to *Plau*, our commercial qPCR arrays also revealed that Thrombospondin-1 (*Thbs1*) was misregulated in *Chd4^fl/fl^;Tie2-Cre^+^* endothelial cells (Table S2). However, unlike *Plau*, which was upregulated in the absence of CHD4, *Thbs1* was significantly downregulated. This result was noteworthy because THBS1 inhibits the ECM protease plasmin as well as matrix metalloproteinases involved in ECM degradation [43], [44]. Therefore, downregulation of *Thbs1* could contribute to the excessive degradation of ECM components seen in our *Chd4^fl/fl^;Tie2-Cre^+^* embryos.

To validate our array data, we performed direct qPCR with *Thbs1*-specific primers and found a significant decrease in the transcript level of *Thbs1* in endothelial cells isolated from *Chd4^fl/fl^;Tie2-Cre^+^* embryos (Figure 3A). *Thbs1* transcripts were similarly decreased in CHD4 knockdown C166 endothelial cells (Figure 3C). Immunostaining confirmed that THBS1 protein expression was diminished around the dorsal aortae of E9.5 *Chd4^fl/fl^;Tie2-Cre^+^* embryos (Figure S8D and S8F). Furthermore, the matrix metalloproteinase MMP9—which is normally inhibited by THBS1 [44]—was upregulated around E10.5 *Chd4^fl/fl^;Tie2-Cre^+^* dorsal aortae (Figures S8H and S8J). ChIP assays revealed that CHD4 and HDAC1 associated with the *Thbs1* promoter approximately 800 bp upstream of the transcription start site (Figure 3D). Furthermore, overexpression of CHD4 in C166 endothelial cells resulted in significantly increased *Thbs1* transcription, as measured by qPCR (Figure 3E). Altogether our data indicated that CHD4 directly promoted *Thbs1* expression in endothelial cells.

To examine the mechanism by which CHD4 promoted *Thbs1* expression in endothelial cells, we assessed histone acetylation by ChIP in the region of the *Thbs1* promoter where CHD4 and HDAC1 bound. As with the *Plaur* promoter, we saw no changes in H3K9 or H3K27 acetylation upon CHD4 knockdown (Figure S7). Therefore, the mechanism by which CHD4-containing NuRD complexes promote *Thbs1* expression in endothelial cells remains unknown.

### Plasmin Activity Is High around Rupture-Prone *Chd4^fl/fl^;Tie2-Cre^+^* Vessels

**Figure 4:**
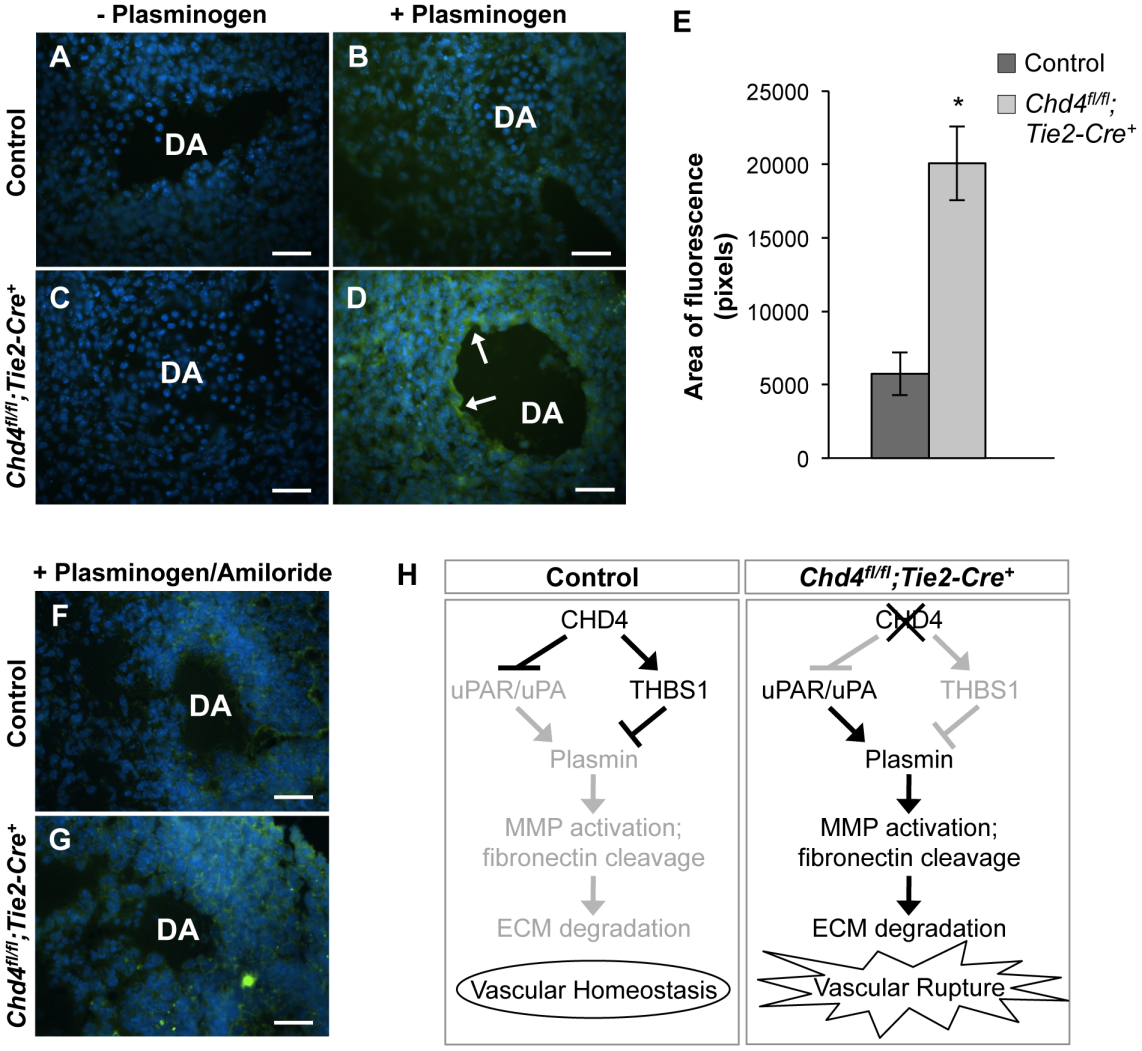
Excessive plasmin activity is detected around rupture-prone *Chd4^fl/fl^;Tie2-Cre^+^* blood vessels. (A–D) Representative images of E10.5 littermate control (A,B) and *Chd4^fl/fl^;Tie2-Cre^+^* (C,D) dorsal aortae (DA) subjected to in situ zymography for detection of plasmin activity. Unfixed sections were overlaid with quenched fluorescent casein, a plasmin substrate that fluoresces upon cleavage. In the presence of exogenous plasminogen, casein cleavage (green fluorescence) was substantially higher around *Chd4^fl/fl^;Tie2-Cre^+^* versus control dorsal aortae (arrows in D versus B). The lack of significant casein cleavage in the absence of exogenous plasminogen (A and C) indicates that the casein cleavage seen in D resulted from plasmin activity. Hoechst (blue) was used for counterstaining. (E) Quantification of three independent in situ zymography experiments such as those shown in B and D from three sets of control and *Chd4^fl/fl^;Tie2-Cre^+^* embryos. Fluorescence generated by casein cleavage was quantified and measured in pixels, comparing comparably sized control and *Chd4^fl/fl^;Tie2-Cre^+^* dorsal aortae. Data are presented as mean ± SD. (F–G) In situ zymography for plasmin activity was performed as described in A for control and *Chd4^fl/fl^;Tie2-Cre^+^* embryonic sections in the presence of the uPA inhibitor amiloride. Amiloride decreased the level of plasmin activity surrounding the *Chd4^fl/fl^;Tie2-Cre^+^* DA (G) to that seen surrounding the control DA (F). (H) Model for how CHD4 impacts ECM proteolysis and vascular integrity through transcriptional regulation of *Thbs1* and *Plaur*. (Left panel) In control endothelial cells, plasmin production and ECM degradation are curbed by CHD4-mediated inhibition of the genes encoding uPA/uPAR and activation of the gene encoding THBS1, resulting in vascular homeostasis. (Right panel) In *Chd4^fl/fl^;Tie2-Cre^+^* endothelial cells, loss of CHD4 leads to increased plasmin activation, which enhances MMP activation and fibronectin cleavage. The net result is excessive ECM degradation and vascular rupture. Scale bars: 100 µm.

Since uPAR and THBS1 respectively promote and inhibit activation of the ECM protease plasmin [43], [45], we hypothesized that upregulated uPAR/uPA and downregulated THBS1 activities led to excessive plasmin activation around rupture-prone *Chd4^fl/fl^;Tie2-Cre^+^* vessels. To test this hypothesis, we conducted in situ zymography assays on sections of E10.5 control and *Chd4^fl/fl^;Tie2-Cre^+^* embryos (Figure 4A–4D). Plasmin activity within tissue sections was detected with the quenched fluorescent plasmin substrate casein, which fluoresces upon cleavage. After addition of the plasmin zymogen plasminogen, which normally circulates in plasma but must be added exogenously to tissue sections, plasmin activity was significantly elevated around *Chd4^fl/fl^;Tie2-Cre^+^* versus control dorsal aortae (Figure 4D versus Figure 4B; quantification in Figure 4E). These results indicated that proteolytic activators of plasmin were upregulated around rupture-prone *Chd4^fl/fl^;Tie2-Cre^+^* blood vessels. Elevated plasmin activity was also detected in E10.5 *Chd4^fl/fl^;Tie2-Cre^+^* hearts (Figure S9), which may explain the slight hypotrabeculation seen in mutant hearts (Figure S3). We did not see elevated plasmin activity around microvessels in mutant limb buds (data not shown), which was consistent with the undiminished ECM components we observed around these vessels and our finding that they were not prone to rupture. Therefore, although CHD4 is expressed in endothelium lining both large and small vessels (Figure S1), it appears to regulate plasmin production preferentially around large truncal vessels that are subject to higher hemodynamic forces at midgestation.

Plasmin can be activated by two independent triggers: uPA or the tissue-type plasminogen activator (tPA). We assessed expression of tPA (*Plat*) by qPCR in CHD4 knockdown C166 endothelial cells to determine if tPA—like uPA—was aberrantly upregulated in the absence of CHD4. We did not see significant chances in *Plat* transcription (Figure S10). To further confirm that elevated uPA triggered the excessive plasmin activity seen around rupture-prone *Chd4^fl/fl^;Tie2-Cre^+^* dorsal aortae, we performed in situ zymography assays on sections of control and mutant embryos in the presence of the uPA-specific inhibitor amiloride, which does not inhibit tPA activity [46]. Amiloride treatment caused a dramatic decrease in the amount of plasmin activity around *Chd4^fl/fl^;Tie2-Cre^+^* dorsal aortae (Figure 4G versus 4D). These data indicated that elevated plasmin activity around rupture-prone *Chd4^fl/fl^;Tie2-Cre^+^* vessels resulted from upregulated uPA. We suspected that this elevated plasmin activity was responsible for the ECM degradation and vascular rupture seen in *Chd4^fl/fl^;Tie2-Cre^+^* embryos (Figure 4H), since excessive plasmin production is associated with vessel wall destruction and aneurysm formation in adult mice [47].

### Genetic Reduction of Urokinase Partially Rescues *Chd4^fl/fl^;Tie2-Cre^+^* Vascular Rupture at E11.5

**Figure 5:**
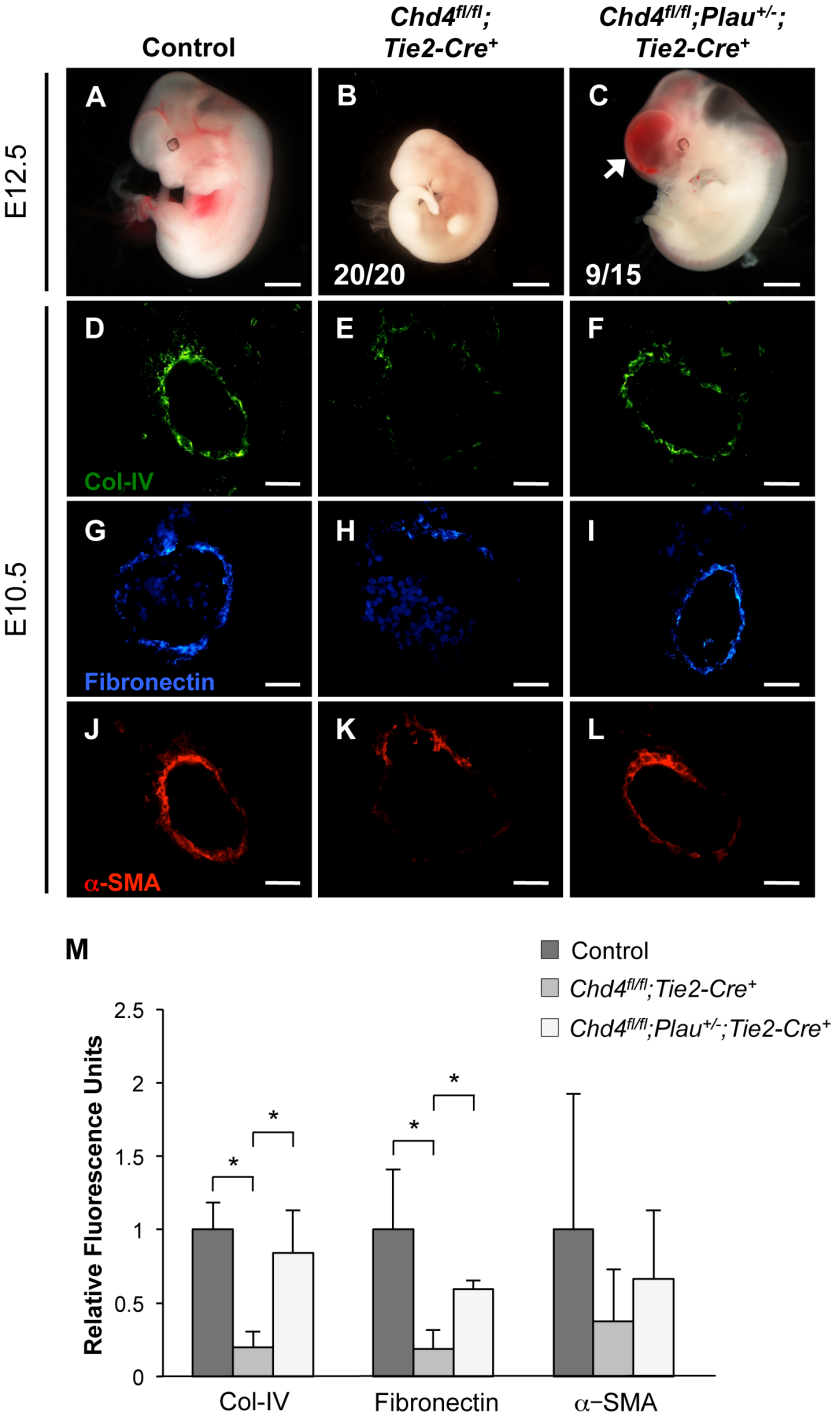
Genetic reduction of urokinase restores ECM components and partially rescues vascular rupture in *Chd4^fl/fl^;Tie2-Cre^+^* embryos. (A–C) Representative images of littermate control (A), *Chd4^fl/fl^;Tie2-Cre^+^* (B), and *Chd4^fl/fl^;Plau^+/−^;Tie2-Cre^+^* (C) embryos at E12.5. All *Chd4^fl/fl^;Tie2-Cre^+^* embryos examined (20/20) were pale and necrotic (B). 60% of *Chd4^fl/fl^;Plau^+/−^;Tie2-Cre^+^* embryos (9 out of 15) were comparable in size to littermate control embryos, although they displayed blood in the brain (arrow) and/or spinal cord (C). (D–L) Histological sections of dorsal aortae from E10.5 control, *Chd4^fl/fl^;Tie2-Cre^+^*, and *Chd4^fl/fl^;Plau^+/−^;Tie2-Cre^+^* embryos were stained with antibodies against type IV collagen (Col-IV; D–F), fibronectin (G–I), or α-SMA (J–L). Scale bars: 1 mm (A–C); 50 µm (D–L). (M) Quantification of immunostained ECM components, such as those shown in panels D–L. Relative fluorescent intensity was measured and normalized to fluorescence in the control sections. Error bars represent SD of results from three independent experiments using three different sets of littermate embryos. Significant differences were calculated using a two-tailed Student's *t* test (*, *p*<0.05).

Since urokinase (uPA) is a potent activator of plasmin and is upregulated in *Chd4^fl/fl^;Tie2-Cre^+^* endothelial cells, we predicted that genetic reduction of uPA (*Plau*) could rescue *Chd4^fl/fl^;Tie2-Cre^+^* embryos from vascular rupture. *Plau^−/−^* mice bred poorly in our colony, so we crossed *Chd4^fl/fl^;Tie2-Cre^+^* embryos onto a *Plau^+/−^* background. We dissected embryos at E12.5 to determine whether urokinase reduction could rescue the vascular rupture typically seen in *Chd4^fl/fl^;Tie2-Cre^+^* embryos at E11.5. At E12.5, all the *Chd4^fl/fl^;Tie2-Cre^+^* embryos we assessed (N = 20) were severely necrotic and partially resorbed (Figure 5B). By contrast, 60% of *Chd4^fl/fl^;Plau^+/−^;Tie2-Cre^+^* embryos (9 out of 15) were comparable in size to littermate controls and showed no evidence of necrosis (Figure 5A and 5C). Additionally, dorsal aortae within these visibly rescued *Chd4^fl/fl^;Plau^+/−^;Tie2-Cre^+^* embryos were intact and appeared histologically comparable to control dorsal aortae at E12.5 (Figure S11A and S11B). However, although *Chd4^fl/fl^;Plau^+/−^;Tie2-Cre^+^* embryos bypassed the dorsal aorta/cardinal vein rupture consistently seen in *Chd4^fl/fl^;Tie2-Cre^+^* embryos at E11.5, many *Chd4^fl/fl^;Plau^+/−^;Tie2-Cre^+^* embryos displayed blood in their brain ventricles and in the central canal of their spinal cords (Figure 5C and Figure S11D). Therefore CHD4 may play a role in central nervous system vascular formation or maintenance at E12.5. Nevertheless, these rescue data demonstrated that urokinase activity contributed to *Chd4^fl/fl^;Tie2-Cre^+^* vascular rupture and lethality at E11.5.

### Genetic Reduction of Urokinase Rescues Expression of ECM Components around *Chd4^fl/fl^;Tie2-Cre^+^* Vessels

Since urokinase reduction partially rescued vascular rupture and lethality in E11.5 *Chd4^fl/fl^;Tie2-Cre^+^* embryos, we questioned whether it also rescued ECM components around rupture-prone vessels. By immunostaining, we saw a significant rescue of type IV collagen and fibronectin expression levels around E10.5 *Chd4^fl/fl^;Plau^+/−^;Tie2-Cre^+^* versus *Chd4^fl/fl^;Tie2-Cre^+^* dorsal aortae (Figure 5D–5I and Figure 5M). We likewise saw an increase in α-SMA-positive cells around *Chd4^fl/fl^;Plau^+/−^;Tie2-Cre^+^* dorsal aortae compared to *Chd4^fl/fl^;Tie2-Cre^+^* dorsal aortae (Figure 5J–5M). These findings supported our central hypothesis that misregulation of uPA/uPAR activity resulted in excessive ECM proteolysis that contributed to *Chd4^fl/fl^;Tie2-Cre^+^* vascular rupture and lethality at E11.5.

## Discussion

The extracellular matrix plays a key role in supporting vascular integrity, however little is known about how ECM production and degradation are regulated and coordinated during embryonic blood vessel development. The first evidence that epigenetic factors could impact embryonic vascular integrity through regulation of ECM composition came from genetic deletion of the histone deacetylase HDAC7. Conditional deletion of *Hdac7* in endothelial cells results in lethal vascular rupture and hemorrhage by E11.5 associated with upregulated expression of the matrix metalloproteinase *Mmp10* [8]. The ATP-dependent chromatin-remodeling enzyme BRG1 similarly regulates endocardial expression of the secreted matrix metalloproteinase *Adamts1*, although vascular *Brg1* mutants suffer from defective cardiac trabeculation rather than vascular rupture due to increased ADAMTS1 activity [25]. Our work now builds on these precedents by demonstrating that ATP-dependent chromatin-remodeling complexes also contribute to the regulation of embryonic vascular integrity by modulating expression of ECM regulators in endothelial cells. We show that NuRD coordinates differential expression of at least two genes to achieve a common outcome of ECM homeostasis. Importantly, genetic reduction of ECM proteolysis partially rescues vascular rupture in our NuRD-deficient mutants, providing convincing evidence that NuRD promotes ECM stability to maintain vascular integrity.

We hypothesize that excessive plasmin production contributes significantly to *Chd4^fl/fl^;Tie2-Cre^+^* vascular rupture. Importantly, we did not see evidence of significant plasmin generation around large or small vessels in control embryos by in situ zymography, suggesting that plasmin levels are normally low at midgestation. Plasmin mediates basement membrane degradation required for sprouting angiogenesis in maternal vessels during embryonic implantation and in tumor vessels during neovascularization [48], [49]. However, it is questionable whether plasmin plays a comparable role during embryonic angiogenesis. Knockouts for various plasmin-generating components such as plasminogen (*Plg*), uPA (*Plau*), and uPAR (*Plaur*) all survive development without notable angiogenesis defects [50]–[53]. Moreover, plasmin-mediated activation of the matrix metalloproteinases MMP1 and MMP10 causes vascular regression rather than sprouting *in vitro* [54]–[56]. These findings are supported by the report that excessive activation of MMP10 *in vivo* results in vascular rupture similar to that seen in our *Chd4^fl/fl^;Tie2-Cre^+^* embryos [8]. Therefore, plasmin may not promote vessel formation in the midgestation embryo but rather may trigger detrimental vessel breakdown by activation of pro-regressive proteinases. If true, minimization of plasmin production or activity—particularly in large vessels that are subject to high hemodynamic forces and increased fragility—would be desirable during embryonic development.

In adult tissues excessive plasmin generation and activity are curtailed by an anti-plasmin system that includes the plasminogen activator inhibitors 1 and 2 (PAI1/2) and α2-antiplasmin. However, expression levels and activities of these inhibitors in the midgestation embryo are unclear. Mice genetically depleted of PAI-1 (*Serpine1*), PAI-2 (*Serpinb2*), or α2-antiplasmin (*Serpinf2*)—either alone or in combination—survive development without hemorrhagic events [57]–[59]. These findings suggest that the traditional anti-plasmin system is not required for suppressing excessive plasmin production and protecting vascular integrity during embryonic development. Instead, we propose that a chromatin-based mechanism for regulating transcription of plasminogen activators may have evolved to limit plasmin production prior to the developmental time point at which a robust anti-plasmin system is established.

Plasmin directly cleaves the ECM components laminin and fibronectin and activates multiple matrix metalloproteinases (MMPs) that degrade the ECM components collagen and elastin [60]. One of the MMPs activated by plasmin, MMP9, is directly inhibited by THBS1 [44]. Therefore, THBS1 may influence ECM proteolysis and vascular integrity during development by inhibiting both plasmin and MMP9. In addition, THBS1 is a chemoattractant for vascular smooth muscle cells [61], so diminished *Thbs1* expression in *Chd4^fl/fl^;Tie2-Cre^+^* endothelial cells may contribute to the reduced α-SMA staining and the disrupted attachments we saw between smooth muscle cells and endothelial cells around mutant vessels. Given the multiple ways that THBS1 can impact ECM stability and vascular integrity, it is possible that the unexplained 25% embryonic lethality observed in *Thbs1^−/−^* mice [62] may be due to vascular rupture and lethal hemorrhage such as that seen in *Chd4^fl/fl^;Tie2-Cre^+^* embryos. We suspect the partial rescue of vascular rupture seen in *Chd4^fl/fl^;Plau^+/−^;Tie2-Cre^+^* embryos would be more penetrant if this cross were able to rescue additional functions of THBS1 beyond plasmin inhibition.

It is also likely that CHD4/NuRD has other primary or secondary genomic targets beyond *Thbs1* and *Plaur* whose misregulation contribute to the vascular rupture phenotype seen in *Chd4^fl/fl^;Tie2-Cre^+^* embryos. For example, integrin β3 (*Itgb3*) was significantly downregulated in *Chd4^fl/fl^;Tie2-Cre^+^* endothelial cells (Table S2). Integrin β3 mediates interactions between the ECM and endothelial cells [63], and vascular integrity is compromised in *Itgb3^−/−^* mice, which experience postnatal hemorrhage [64]. We could not detect binding of CHD4 to the *Itgb3* promoter by chromatin immunoprecipitation, so we suspect *Itgb3* downregulation in *Chd4^fl/fl^;Tie2-Cre^+^* endothelial cells was a secondary consequence of *Chd4* deletion. Nevertheless, decreased *Itgb3* in *Chd4^fl/fl^;Tie2-Cre^+^* endothelial cells may partially contribute to vascular fragility at midgestation. Likewise, it is possible that misregulated Wnt signaling contributes to vascular fragility in *Chd4^fl/fl^;Tie2-Cre^+^* embryos. We had previously determined that the Wnt-responsive transcription factor *Tcf7* and a subset of Wnt target genes—including *Plaur*—are upregulated in *Chd4^fl/fl^;Tie2-Cre^+^* yolk sac endothelial cells [23]. However, this upregulation of Wnt signaling does not appear to impact vascular patterning, as is seen in vascular gain-of-function mutants for the central Wnt signaling component β-catenin (*β-catenin^lox(ex3)/lox(ex3)^;Tie2-Cre^+^*) [65]. This phenotypic dissimilarity may be due to the fact that fewer Wnt target genes are misregulated in *Chd4^fl/fl^;Tie2-Cre^+^* vasculature than in *β-catenin^lox(ex3)/lox(ex3)^;Tie2-Cre^+^* blood vessels. Although *β-catenin^lox(ex3)/lox(ex3)^;Tie2-Cre^+^* embryos show no evidence of vascular rupture or hemorrhage prior to lethality at E11.5 [65], we cannot exclude the possibility that upregulated vascular Wnt signaling might contribute to blood vessel fragility in *Chd4^fl/fl^;Tie2-Cre^+^* embryos.

Finally, it will be important to determine if the increased ECM proteolysis and vascular rupture seen in *Chd4^fl/fl^;Tie2-Cre^+^* embryos are recapitulated in postnatal mice with induced vascular *Chd4* excision. It is possible that plasminogen/plasmin inhibitors such as PAI-1 and α2-antiplasmin reduce the requirement for CHD4 in adult vasculature. However, if CHD4 limits plasmin production in adult endothelium as it does in the embryo, it may play an important preventative role in vascular pathologies such as abdominal aortic aneurysms, which are associated with excessive ECM degradation around vessel walls [66]. uPA/uPAR and plasmin have previously been recognized as promising therapeutic targets for combating aneurysm progression [67], and we now present a novel chromatin-based mechanism by which the activity of these proteins can be regulated during embryonic vascular development. Future studies will determine if NuRD influences proteolytically triggered aneurysms in adults.

## Materials and Methods

### Ethics Statement

The Oklahoma Medical Research Foundation (OMRF) is accredited by AAALAC International and follows the Public Health Service Policy for the Care and Use of Laboratory Animals. Animal care was provided in accordance with the procedures outlined in the Guide for Care and Use of Laboratory Animals (National Academies Press; Washington, D.C.; 1996). The OMRF Institutional Animal Care and Use Committee approved all animal use protocols.

### Mice

*Chd4*-floxed mice (*Chd4^fl/fl^*), *uPA^+/−^* mice, and *Tie2-Cre* transgenic mice have been described [20], [52], [68]. All mice were maintained on a mixed genetic background at the Oklahoma Medical Research Foundation animal facility. *Tie2-Cre* transgenic and *Chd4*-floxed embryos and mice were genotyped as described [23], [69]. *uPA^+/−^* mice and embryos were genotyped by PCR, using primer sequences obtained from Jackson Laboratory. The wildtype *uPA* allele was amplified with a forward primer (5′-TCTGGAGGACCGCTTATCTG-3′) and a reverse primer (5′-CTCTTCTCCAATGTGGGATTG-3′) that yielded a 153 bp amplicon. The targeted *uPA* allele was amplified by a neomycin-specific forward primer (5′-CACGAGACTAGTGAGACGTG-3′) and the same reverse primer used for detecting the wildtype allele, to yield a 337 bp amplicon.

### Histological Staining

Hematoxylin and eosin (H&E) staining was performed on embryo cryosections as described previously [70]. Alcian blue staining was performed on paraffin sections of embryonic hearts. Sections were dewaxed, rehydrated, and stained with a 1% solution of Alcian blue 8 GX (Sigma) in acetic acid for 30 min. After Alcian blue staining, sections were counterstained with Nuclear Fast Red (Vector Laboratories) for 5 min.

### Transmission Electron Microscopy

Tissue samples were fixed by immersion in a mixture of 2% paraformaldehyde and 2.5% glutaraldehyde in 0.1 M sodium cacodylate for 1 h, followed by postfixation in 1% osmium tetroxide for 90 min and 1% tannic acid for 1 h. The samples were subsequently dehydrated in a graded ethanol series and embedded in epoxy resin (Electron Microscopy Sciences). Ultrathin sections (70 nm) were obtained using an RMC 7000 ultramicrotome (Boeckeler Instruments) equipped with a diamond knife. Sections were stained with uranyl acetate and lead citrate before being viewed with a Hitachi H-7600 electron microscope equipped with a 4 megapixel digital monochrome camera and AMT-EM image acquisition software (Advanced Microscopy Techniques).

### Immunohistochemistry

*Chd4^fl/fl^;Tie2-Cre^+^* and littermate control embryos were dissected, fixed in 4% PFA overnight, and passed through 10% sucrose for 10 min, 15% sucrose for 10 min, 20% sucrose for 1 hr and a 1∶1 mixture of 20% sucrose and Optimal Cutting Temperature compound (O.C.T.; Sakura Finetek) overnight. Embryos were embedded in O.C.T. the following morning. Yolk sac tissue was used for genotyping. 8 µm sections were cut with a Microm HM 505 E cryotome (Microm International) and adhered to Superfrost Plus slides (Fisher Scientific). Histological sections were blocked in blocking solution [5% normal goat serum/5% normal donkey serum/0.3% bovine serum albumin/0.1% Triton X-100 in phosphate buffered saline (PBS)] for 2 h at room temperature. Sections were incubated in primary antibody at a 1∶100–1∶500 concentration in blocking solution for 1 h at room temperature, washed three times (3 min each) in 0.1% Triton X-100/PBS, then incubated for 1 h at room temperature in secondary antibody at a 1∶500 concentration in blocking solution along with 20 ug/mL Hoechst stain. Sections were then washed three times (3 min each) in 0.1% Triton X-100/PBS and coverslipped with 2.5% DABCO/90% glycerol/PBS, pH 8.6. Primary antibodies used for immunohistochemistry were rat-anti-PECAM-1 (BD Pharmingen, 553370); rabbit-anti-phospho-histone H3 (Millipore, 06-570); rabbit-anti-Ki67 (Thermo Scientific, RM-9106-SO); Cy3-labeled mouse-anti-α-SMA (Sigma-Aldrich, C6198); rabbit-anti-active-Caspase 3 (Abcam, ab13847); mouse-anti-THBS-1 (Abcam, ab1823); rat-anti-VE-Cadherin (BD Pharmingen, 550548); rabbit-anti-Col-IV (Abcam, ab19808); mouse-anti-Fibronectin (Sigma, F6140); rabbit-anti-MMP9 (Abcam, ab38898); rabbit-anti-CHD4 (Active Motif, 39289); and rabbit-anti-ZO-1 [71]. Secondary antibodies used for immunohistochemistry were Cy3-donkey-anti-rat IgG (Jackson ImmunoResearch), Alexa 488-goat-anti-rabbit IgG (Invitrogen) and Alexa 488-goat-anti-mouse IgG (Invitrogen).

### Cell Culture and Transfections

The C166 murine yolk sac-derived endothelial cell line (ATCC, CRL-2581) was maintained as described [69]. For CHD4 knockdown, cells were transfected with 100 nM CHD4 siGENOME SMARTpool or nontargeting control small interfering RNA (siRNA) oligonucleotides (Dharmacon catalog numbers M-052142-01 or D-001210-01, respectively) or with 100 nM CHD4 Silencer Select or nontargeting control siRNA oligonucleotides (Life Technologies, AM4637 and 4390816, respectively) using Lipofectamine 2000 (Invitrogen) in serum-free OptiMEM (Invitrogen). After 24 h, cells were harvested in Laemmli buffer (62.5 mM Tris, pH 6.8, 10% glycerol, 5% SDS, 0.01% bromophenol blue) for Western blot analysis or TRIzol (Invitrogen) for transcript analysis. For transfection with the CHD4 expression plasmid (Fisher, MMM1013-202770503) or with its analogous empty vector backbone, which was generated by removing CHD4 with SalI and NotI restriction enzymes, C166 cells were transfected as above with 0.02 ng of plasmid for 24 h.

### Western Blot

Total protein was harvested from siRNA-transfected C166 cells, fractionated in a 9% SDS polyacrylamide gel, and transferred to a PVDF membrane for Western blot analysis with antibodies to CHD4 (Abcam, ab72418) and GAPDH (Sigma, G9545).

### Luciferase Assays

A fragment of the murine *Plaur* promoter spanning the transcription start site (−952 bp to +303 bp) was amplified by PCR and cloned into a modified chromatinizable pCEP9 vector (gift of Jesse Raab). The luciferase gene from a pGL3 luciferase reporter vector was also cloned into the pCEP9 vector. The complete *Plaur* promoter reporter was transfected into C166 endothelial cells (250 ng) using Lipofectamine 2000. A renilla luciferase plasmid (pRL-TK) was co-transfected (10 ng) as a control. Reporter activity was assayed by transfection of 10 pmol NS siRNA or CHD4 siRNA oligonucleotides (Life Technologies, AM4637 and 4390816, respectively) or with 20 ng of the CHD4 expression plasmid (or analogous control plasmid) described above in “cell culture and transfections.” Twenty-four hours after transfection, cells were harvested and analyzed with the Dual-Luciferase Reporter Assay System (Promega) and a Glomax 20/20 Luminometer (Promega).

### Fluorescence Microscopy and Image Acquisition

Gross embryonic images were obtained with a Nikon SMZ800 stereomicroscope and Nikon DS-Fi1 camera and monitor. Brightfield histological images were obtained with a Nikon Eclipse 80i microscope using a 10× (NA 0.3) and 40× (NA 0.75) objective and a Nikon DS-Fi1 camera. Fluorescent images were obtained with a Nikon Eclipse 80i microscope using 10× (NA 0.3), 20× (NA 0.5), and 40× (NA 0.75) objectives, an X-cite 120Q light source, and a Nikon DS-Qi1Mc camera. NIS-Elements AR3.0 (Nikon) software was used for all brightfield and fluorescent image acquisition and assembly. Relative fluorescence was determined using ImageJ software (National Institutes of Health).

### Confocal Microscopy and Image Acquisition

Confocal images for VE-Cadherin and ZO-1 immunostained sections were obtained on an Olympus IX81 motorized inverted microscope with a Lumens 200 light source. Z stacks were acquired using an Olympus DSU spinning disc confocal system and analyzed using Slidebook 5.0 imaging software (Intelligent Imaging Innovations).

### In Situ Zymography

E10.5 embryos were cryosectioned without prior fixation. 20 µm sections of control and *Chd4^fl/fl^;Tie2-Cre^+^* embryos were affixed to the same slide and were overlaid with in situ zymography solution (ISZS) consisting of 1% low melting point agarose (Invitrogen), 0.1 mg/mL quenched BODIPY FL casein from the EnzChek Protease Assay Kit (Invitrogen), 1 U/mL human Glu-plasminogen (Sigma), and Hoechst (20 µg/mL). Overlaid sections were coverslipped and incubated for 1 h at 37°C. Casein cleavage was detected by fluorescence microscopy. Plasminogen-free ISZS was used as a negative control to verify that casein cleavage was plasmin-dependent. For amiloride treatment, 0.1 mM amiloride (Sigma, A7410) was mixed with the ISZS prior to overlaying embryo sections.

### Primary Endothelial Cell Isolation

E10.5 embryos were digested with collagenase-DNase solution [1.5 mg/mL collagenase, 25 mg/mL DNase, 25 mM HEPES in Dulbecco's Modified Eagle Medium (DMEM)] for 30 min at 37°C. Cells were washed once with PBS/0.1% BSA and resuspended in DMEM. Dynabeads (Life Technologies) conjugated to PECAM-1 antibody (BD Pharmingen, 557355) were added, and samples were incubated for 30 min at 4°C with rotation. Immunoprecipitated cells were washed once with PBS/0.1% BSA and eluted in Trizol (Life Technologies).

### qPCR

To analyze transcript levels, total RNA isolated from primary vascular endothelial cells was purified using the RNeasy Mini Kit (QIAGEN) according to the manufacturer's instructions. cDNA was prepared using the MultiScribe Reverse Transcriptase kit (Applied Biosystems), and real-time quantitative PCR was performed using 2× SYBR green qPCR master mix (Life Technologies) and the CFX96 Real-Time System (Bio-Rad) with gene-specific primers. The following primers were used for qPCR: *Chd4* (5′-TCCTCTGTCCACCATCATCA-3′ and 5′-ACCCAAGATGGCCATATCAA-3′); *Plau* (5′-GCGCCTTGGTGGTGAAAAAC-3′ and 5′-TTGTAGGACACGCATACACCT-3′); *Plaur* (5′-GGCTTAGATGTGCTGGGAAA-3′ and 5′-CAATGAGGCTGAGTTGAGCA-3′); *Thbs1* (5′-CCAAAGCCTGCAAGAAAGAC -3′ and 5′-CCTGCTTGTTGCAAACTTGA-3′); *Hbegf* (5′-GACCCATGCCTCAGGAAATA-3′ and 5′-GGCATTTGCAAGAGGGAGTA-3′); *Pdgfb* (5′-CTGCTGCAATAACCGCAAT-3′ and 5′-CCGAGGGGTCACTACTGTCT-3′); *Tgfbr2* (5′-GCAAGTTTTGCGATGTGAGA-3′ and 5′-GGCATCTTCCAGAGTGAAGC-3′); *Plat* (5′-CTGAGGTCACAGTCCAAGCA-3′ and 5′-TCAGCCGGTCAGAGAAGAAT-3′); *Gapdh* (5′-TCAACGGCACAGTCAAGG-3′ and 5′-ACTCCACGACATACTCAGC-3′); and *β-actin* (5′-TGTTACCAACTGGGACGACA-3′ and 5′-GGGGTGTTGAAGGTCTCAAA-3′).

### qPCR Analysis

The relative fold change in transcription was determined using the comparative C_T_ method and the housekeeping genes, *Gapdh* and β*-actin*, as internal controls. Data from at least 3 independent sets of littermate control and mutant embryos or from 3 independent siRNA knockdown experiments or overexpression experiments were combined and presented as the mean plus standard deviation (SD). Statistical differences were detected using a two-tailed unpaired Student's *t* test.

### Angiogenesis and Extracellular Matrix qPCR Arrays

Total RNA from primary vascular endothelial cells was isolated using Trizol (Life Technologies) according to the manufacturer's instructions. The DNA-*free* kit (Life Technologies) was used to digest any contaminating DNA, and cDNA was prepared using the RT^2^ First Strand kit (SABiosciences/QIAGEN). Three independent experiments were performed using Mouse Angiogenesis RT^2^ Profiler PCR Array and Mouse Extracellular Matrix and Adhesion Molecules RT^2^ Profiler PCR Array (SABiosciences/QIAGEN) according to the manufacturer's instructions. Data analysis was performed using the web-based PCR array data analysis tool available through the SABiosciences/QIAGEN website.

### Chromatin Immunoprecipitation (ChIP)

Sub-confluent C166 yolk sac endothelial cells were crosslinked with 1% formaldehyde for 10 min and processed for ChIP with the MAGnify Chromatin Immunoprecipitation System (Invitrogen) according to the manufacturer's instructions. A CHD4-specific antibody (Abcam; ab70469) was used to immunoprecipitate protein-DNA complexes, and a normal mouse IgG antibody (Invitrogen; 100005291) was used as a negative control for CHD4 ChIP assays. An HDAC1-specific antibody (Abcam, ab46985) and normal mouse IgG antibody were used for HDAC1 ChIP assays. For H3K9Ac and H3K27Ac ChIP assays, chromatin was harvested from C166 cells that were transfected for 24 h with nonspecific or CHD4-specific siRNAs. Sonicated chromatin was immunoprecipitated using an anti-histone H3K9Ac antibody (Active Motif; 39137) or an anti-histone H3K27Ac antibody (Abcam; ab4729), and isotype matched IgG antibodies (Invitrogen) were used as negative controls. All ChIP assays were performed with 10 µg of experimental or control antibodies. Real-time quantitative PCR was performed using RT^2^ Fast SYBR green qPCR master mix (SABiosciences) and the CFX96 detection system (Bio-Rad) with specific primers. For the *Thbs1* gene, the primers 5′-AGCCAGATGGTTCAGCAAAT-3′ and 5′-CCCTCAAGGCAAAATTGAAA-3′ (located 834 bp and 732 bp upstream of the TSS, respectively) were used for CHD4, HDAC1, H3K9Ac and H3K27Ac ChIP assays. For the *Plaur* gene, the primers 5′-ACTGAGCCGCTCTGAGTGAT-3′ and 5′-CCAGGGGAAAAACAAGTTGA-3′ (located 922 bp and 723 bp upstream of the TSS, respectively) were used for the ChIP assays described above. For the *Plau* gene, the primers 5′-GACATGTGGGAGCCTTTGTT-3′ and 5′-CTCGCTGATCTCAAGCCTCT-3′ (located 507 bp and 349 bp upstream of the TSS, respectively) were used for CHD4 and HDAC1 ChIP assays. Primers designed for a transcriptionally inactive region far upstream of the *Fzd5* TSS were used to amplify a negative control region for CHD4 binding and activity. These *Fzd5UP* primers, 5′-GGTGACTTAGGGCAAAACCA-3′ and 5′-AGGCCACCATACCAGGTTCT-3′, were located 4856 bp and 4648 bp upstream of the TSS, respectively.

### ChIP Analysis

A defined fraction of input chromatin that was subjected to sonication was set aside prior to immunoprecipitation but was otherwise treated identically to immunoprecipitated samples through reverse crosslinking and DNA purification steps. DNA from input chromatin was diluted and analyzed by qPCR along with DNA from immunoprecipitated samples. Input C_T_ values were adjusted for dilution and used to calculate % input values for immunoprecipitated samples. A two-tailed Student's *t* test was used for statistical comparisons between samples immunoprecipitated with negative control (i.e. IgG) or experimental antibodies at a particular locus. Comparisons between immunoprecipitated samples at an experimental locus (i.e. *Plaur* or *Thbs1*) versus a negative control locus (i.e. *Fzd5UP*) were also analyzed.

### Whole-Mount Yolk Sac Staining

Embryos with attached yolk sacs were dissected from maternal tissue, fixed, permeabilized and immunostained with rat anti-mouse PECAM-1 antibody (BD Pharmingen, 557355) as described [69]. Vitelline vessel diameters were measured using NIS-Elements AR3.0 (Nikon) software.

### Whole-Mount Embryo Staining

Whole-mount immunostaining for PECAM-1 was performed using a rat anti-mouse PECAM-1 antibody (BD Pharmingen, 557355) as described [72].

## Supporting Information

Figure S1 CHD4 is expressed in endothelial cells lining large and small vessels at E10.5. Immunostaining for the endothelial cell marker PECAM-1 (red) and for CHD4 (green) was performed on sections of E10.5 wildtype embryos. Hoechst (blue) was used to counterstain nuclei. (A) Many endothelial cells lining the dorsal aorta (DA) display positive CHD4 staining. (B) Many endothelial cells lining capillaries within the limb bud (LB) likewise display positive CHD4 staining. Arrows in the magnified insets indicate individual endothelial cells. Scale bars: 100 µm.
(TIF)

Figure S2 *Chd4^fl/fl^;Tie2-Cre^+^* embryos display normal vascular patterning at E10.5. (A–J) E10.5 littermate control and *Chd4^fl/fl^;Tie2-Cre^+^* embryos (A–F) and yolk sacs (G–J) were whole-mount immunostained with anti-PECAM-1 to visualize vascular patterning. Magnified views of cranial vessels (C,D), intersomitic vessels (E,F), and yolk sac vessels (I,J) are shown. V.V. = yolk sac vitelline vessel. Scale bars: 1 mm (A–B); 100 µm (G–J). (K) Mean vitelline vessel diameter measurements from 6 control and 6 *Chd4^fl/fl^;Tie2-Cre^+^* E10.5 yolk sacs. Error bars represent ± SD, and a two-tailed Student's *t* test showed no statistical difference between controls versus mutants.
(TIF)

Figure S3 *Chd4^fl/fl^;Tie2-Cre^+^* hearts have slightly diminished Alcian blue staining and modest hypotrabeculation at E10.5. Comparable sections of E10.5 littermate control (A,C) and *Chd4^fl/fl^;Tie2-Cre^+^* (B,D) hearts were stained with Alcian blue to detect acidic glycosaminoglycans that contribute to cardiac ECM. Arrow in (B) indicates an area of decreased Alcian blue staining in a mutant ventricle. Arrow in (D) indicates a hypotrabeculated region of a mutant ventricle that also has diminished Alcian blue staining. Images are representative of results from comparisons between 3 littermate control and mutant embryos. Scale bars: 100 µm.
(TIF)

Figure S4 Endothelial cell proliferation and apoptosis are normal in *Chd4^fl/fl^;Tie2-Cre^+^* rupture-prone vessels. (A–H) Histological sections of dorsal aortae (DA) from E10.5 littermate control (A,C,E,G) and *Chd4^fl/fl^;Tie2-Cre^+^* (B,D,F,H) embryos were stained to assess endothelial cell proliferation or apoptosis. Immunostaining with anti-PECAM-1 antibodies (red) was used to visualize the vasculature and Hoechst dye (blue) was used to stain nuclei. Proliferation was evaluated by immunostaining with anti-phosphorylated histone H3 (PPH3; A,B) or anti-Ki67 (C,D) antibodies. Apoptosis was detected by TUNEL staining (E,F) or by immunostaining with anti-active caspase 3 antibodies (G,H). Representative images from three separate experiments are shown; no quantitative differences were detected in staining for PPH3 (0/116 control vs. 0/126 mutant endothelial cells), Ki67 (0/54 control vs. 1/51 mutant endothelial cells), TUNEL (0/136 control vs. 0/121 mutant endothelial cells), or active caspase 3 (1/151 vs. 0/126 endothelial cells). Scale bars: 100 µm.
(TIF)

Figure S5 Control and *Chd4^fl/fl^;Tie2-Cre^+^* endothelial cells lining rupture-prone vessels express comparable levels of intercellular junction markers at E10.5. (A–D) Cryosections of dorsal aortae from E10.5 littermate control (A,C) and *Chd4^fl/fl^;Tie2-Cre^+^* (B,D) embryos were immunostained for the adherens junction marker VE-Cadherin (red; A,B) or the tight junction marker ZO-1 (green; C,D) and analyzed by confocal microscopy. Hoechst (blue) was used as a nuclear counterstain. Representative images from three independent experiments are shown. Scale bars: 10 µm.
(TIF)

Figure S6 Genes involved in smooth muscle cell recruitment toward endothelium are expressed at normal or elevated levels in CHD4 knockdown endothelial cells. C166 endothelial cells were transfected with nonspecific (NS) or CHD4-specific siRNA oligonucleotides for 24 h. RNA was isolated, cDNA was synthesized, and qPCR was performed using gene-specific primers for heparin-binding EGF-like growth factor (*Hbegf*), platelet derived growth factor B (*Pdgfb*), or transforming growth factor β receptor II (*Tgfbr2*). Data were normalized to the relative expression of NS siRNA-treated samples. Error bars represent ± SD of results from three independent experiments. Statistical analysis was performed using a two-tailed Student's *t* test (*, *p*<0.05).
(TIF)

Figure S7 No changes in H3K9Ac or H3K27Ac enrichment are seen at the *Plaur* and *Thbs1* promoters in CHD4 knockdown endothelial cells. Chromatin immunoprecipitation (ChIP) assays were carried out in C166 endothelial cells transfected for 24 h with either non-specific (NS) siRNA or CHD4-specific siRNA oligonucleotides. Immunoprecipitation was performed using antibodies against H3K9Ac (A) or H3K27Ac (B). Immunoprecipitated DNA was analyzed by qPCR to examine enrichment of H3K9Ac or H3K27Ac at the *Plaur* and *Thbs1* promoters. A transcriptionally inactive region approximately 5 kb upstream of the *Fzd5* transcription start site (*Fzd5UP*) was assessed as a negative control for antibody binding. Data are represented as a percent of total input chromatin. Error bars represent SD of results from three independent experiments. For statistical analysis, NS siRNA- and CHD4 siRNA-treated samples were compared at each locus using a two-tailed Student's *t* test; no significant differences were detected. Likewise, no significant differences were detected between NS siRNA- and CHD4 siRNA-treated samples when H3K9Ac and H3K27Ac marks were normalized against total H3 pulldown at the *Plaur* and *Thbs1* promoters (data not shown).
(TIF)

Figure S8 Thrombospondin and its downstream target MMP9 are misexpressed around rupture-prone *Chd4^fl/fl^;Tie2-Cre^+^* dorsal aortae. (A–F) Cryosections of dorsal aortae (DA) from E9.5 littermate control (A,C,E) and *Chd4^fl/fl^;Tie2-Cre^+^* (B,D,F) embryos were stained with anti-PECAM-1 antibodies (red; A,B), anti-THBS1 antibodies (green; C,D), and Hoechst (blue; E,F). Merged images are shown in panels E and F. (G–J) Cryosections of dorsal aortae from E10.5 littermate control (G, I) and *Chd4^fl/fl^;Tie2-Cre^+^* (H,J) embryos were stained with anti-MMP9 antibodies. The boxed regions in panels G and H are magnified in panels I and J, respectively. Scale bars: 50 µm.
(TIF)

Figure S9 Plasmin activity is elevated in E10.5 *Chd4^fl/fl^;Tie2-Cre^+^* hearts. In situ zymography was performed on sections of E10.5 littermate control (A) and *Chd4^fl/fl^;Tie2-Cre^+^* (B) embryonic hearts for detection of plasmin activity, as described in Figure 5. Casein cleavage (green fluorescence) was substantially higher in the *Chd4^fl/fl^;Tie2-Cre^+^* hearts versus the control hearts. Hoechst (blue) was used as a nuclear counterstain. V = ventricle. Scale bars: 100 µm.
(TIF)

Figure S10 Expression of tissue plasminogen activator (tPA; *Plat*) is normal in CHD4 knockdown endothelial cells. C166 endothelial cells were transfected with nonspecific (NS) or CHD4-specific siRNA oligonucleotides for 24 h. RNA was isolated, cDNA was synthesized, and qPCR was performed using *Plat*-specific primers. Data were normalized to the relative expression of NS siRNA-treated samples. Error bars represent ± SD of results from three independent experiments.
(TIF)

Figure S11 Rupture-prone dorsal aortae are significantly rescued from hemorrhage in *Chd4^fl/fl^;Plau^+/−^;Tie2-Cre^+^* embryos. (A–D) Hematoxylin and eosin (H&E) staining of E12.5 littermate control and *Chd4^fl/fl^;Plau^+/−^;Tie2-Cre^+^* embryos revealed an intact dorsal aorta (DA; insets in panels A and B). However, blood was seen aberrantly pooling in the central canal (CC) of the spinal cord in *Chd4^fl/fl^;Plau^+/−^;Tie2-Cre^+^* embryos (D). Scale bars: 500 µm (A–B); 100 µm (C–D).
(TIF)

Table S1 Genes with unchanged expression levels in E10.5 *Chd4^fl/fl^;Tie2-Cre^+^* endothelial cells. Endothelial cells from E10.5 littermate control and *Chd4^fl/fl^;Tie2-Cre^+^* embryos were isolated, RNA was purified, cDNA was synthesized and qPCR was performed using two commercial qPCR arrays containing a total of 157 genes important for extracellular matrix composition and angiogenesis (SABiosciences/QIAGEN). The 148 genes that were identified as having insignificant changes in expression levels (*p*>0.1) after three different experiments are listed. Data analysis was performed using the web-based PCR Array Data Analysis tool recommended for use with these arrays: (http://www.sabiosciences.com/pcrarraydataanalysis.php). See Table S2 for the list of genes with significant expression changes from the arrays.
(DOC)

Table S2 Genes misregulated in E10.5 *Chd4^fl/fl^;Tie2-Cre^+^* embryonic endothelial cells. Endothelial cells from E10.5 littermate control and *Chd4^fl/fl^;Tie2-Cre^+^* embryos were isolated, RNA was purified, cDNA was synthesized and qPCR was performed using two commercial qPCR arrays containing a total of 157 genes important for extracellular matrix composition and angiogenesis (SABiosciences/QIAGEN). The nine genes that were identified as having significantly differential expression levels (*p*<0.1) after three different experiments are listed along with their average fold change. Data analysis was performed using the web-based PCR Array Data Analysis tool recommended for use with these arrays: (http://www.sabiosciences.com/pcrarraydataanalysis.php). See Table S1 for the list of genes with insignificant expression changes from the arrays.
(DOC)
