# Supplementary figures and images for: The NuRD Chromatin-Remodeling Enzyme CHD4 Promotes Embryonic Vascular Integrity by Transcriptionally Regulating Extracellular Matrix Proteolysis
Source: PLoS Genet. 2013 Dec 12;9(12):e1004031. doi: 10.1371/journal.pgen.1004031 (PMC3861115; doi:10.1371/journal.pgen.1004031)

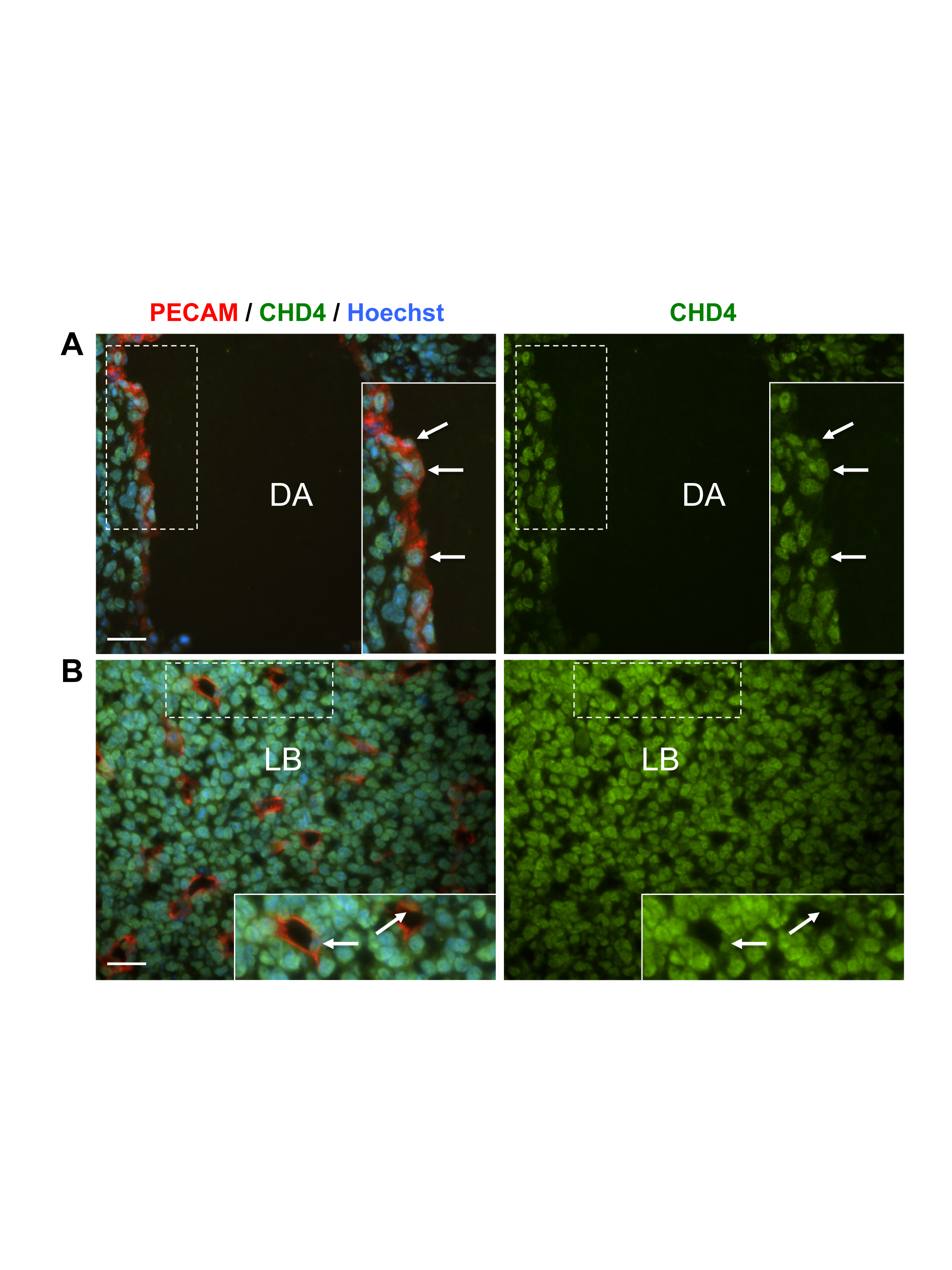

Supplement: Figure S1 — CHD4 is expressed in endothelial cells lining large and small vessels at E10.5. Immunostaining for the endothelial cell marker PECAM-1 (red) and for CHD4 (green) was performed on sections of E10.5 wildtype embryos. Hoechst (blue) was used to counterstain nuclei. (A) Many endothelial cells lining the dorsal aorta (DA) display positive CHD4 staining. (B) Many endothelial cells lining capillaries within the limb bud (LB) likewise display positive CHD4 staining. Arrows in the magnified insets indicate individual endothelial cells. Scale bars: 100 µm. (TIF) [file pgen.1004031.s001.tif]

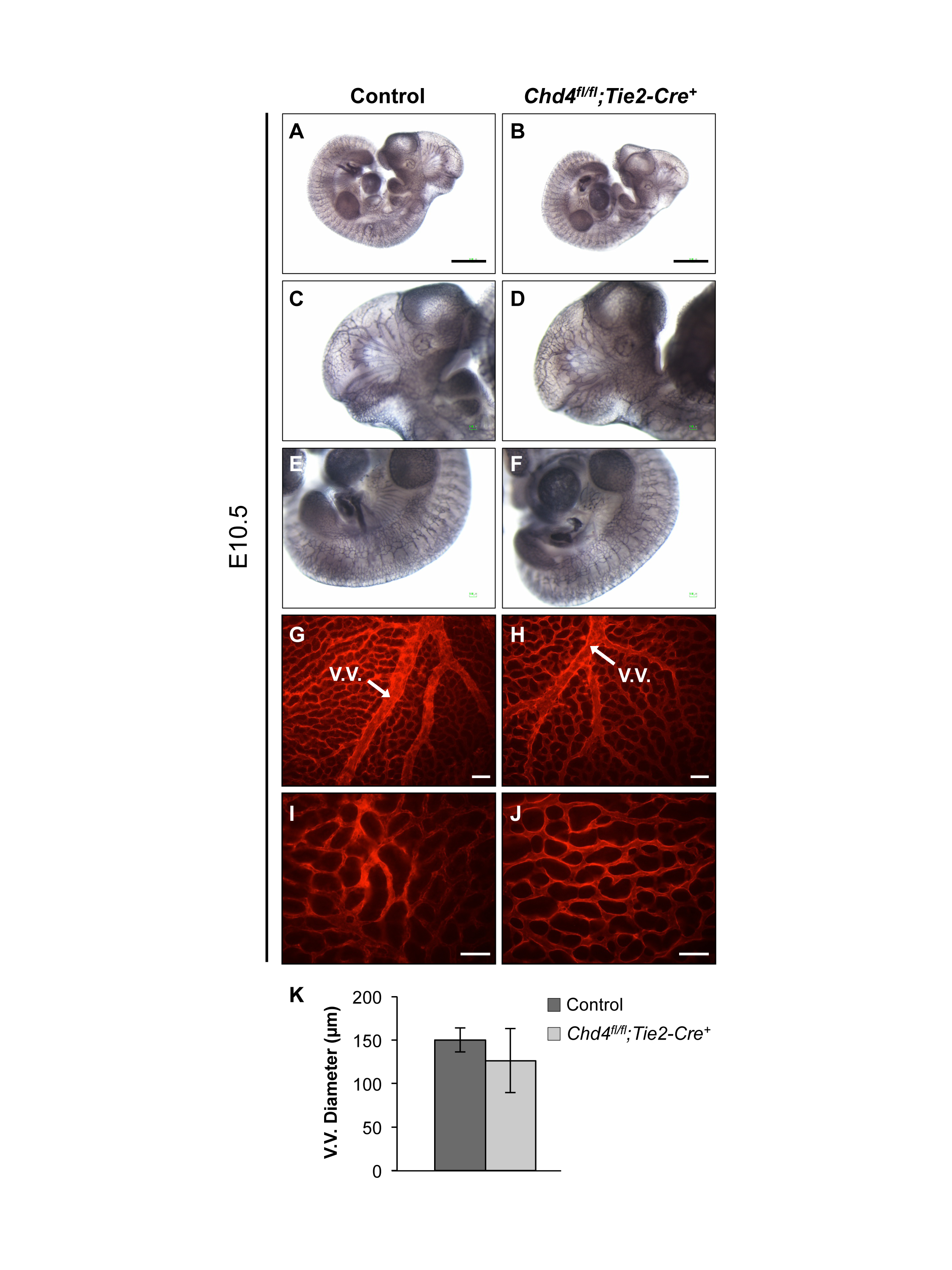

Supplement: Figure S2 — Chd4fl/fl;Tie2-Cre+ embryos display normal vascular patterning at E10.5. (A–J) E10.5 littermate control and Chd4fl/fl;Tie2-Cre+ embryos (A–F) and yolk sacs (G–J) were whole-mount immunostained with anti-PECAM-1 to visualize vascular patterning. Magnified views of cranial vessels (C,D), intersomitic vessels (E,F), and yolk sac vessels (I,J) are shown. V.V. = yolk sac vitelline vessel. Scale bars: 1 mm (A–B); 100 µm (G–J). (K) Mean vitelline vessel diameter measurements from 6 control and 6 Chd4fl/fl;Tie2-Cre+ E10.5 yolk sacs. Error bars represent ± SD, and a two-tailed Student's t test showed no statistical difference between controls versus mutants. (TIF) [file pgen.1004031.s002.tif]

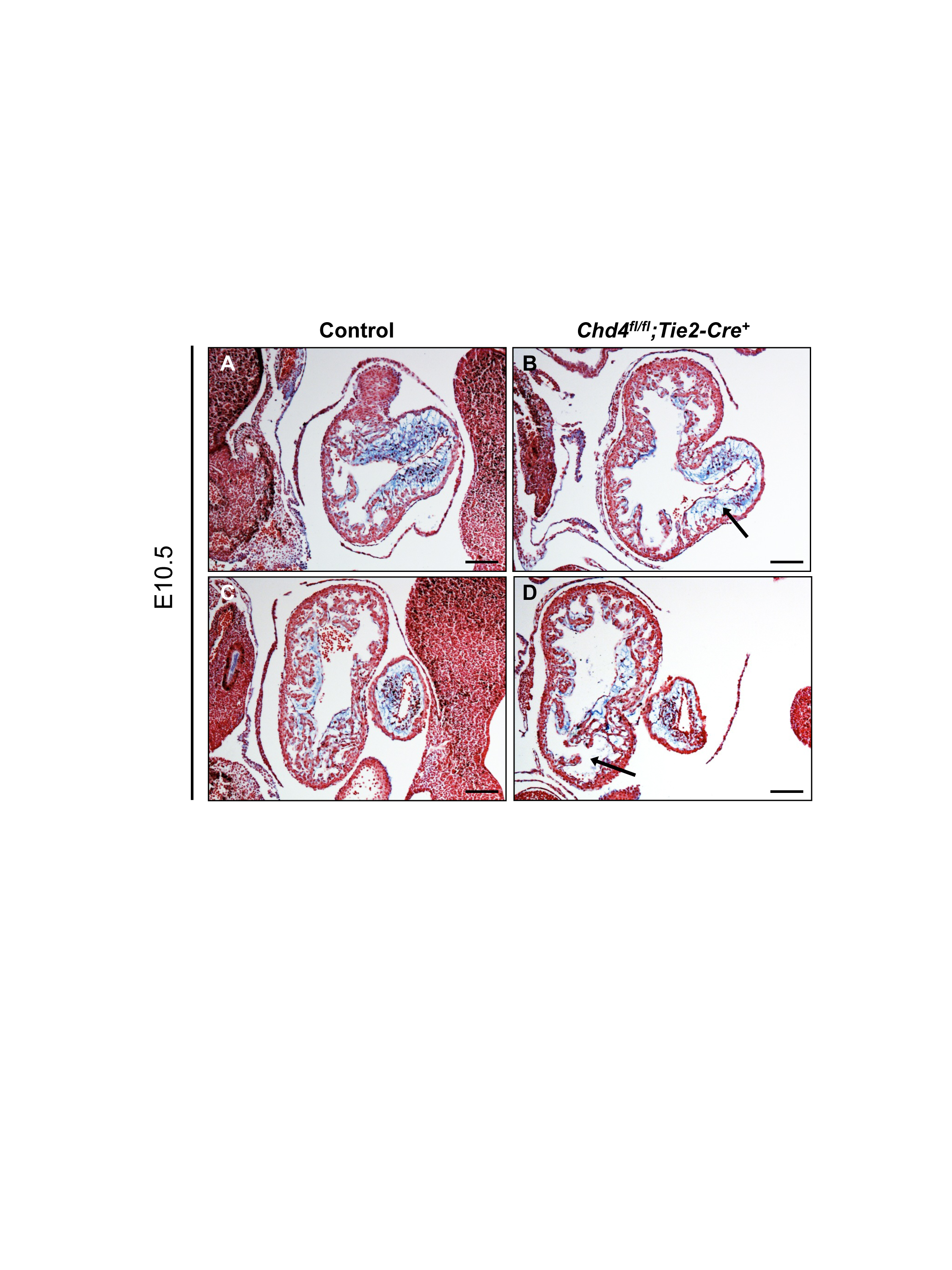

Supplement: Figure S3 — Chd4fl/fl;Tie2-Cre+ hearts have slightly diminished Alcian blue staining and modest hypotrabeculation at E10.5. Comparable sections of E10.5 littermate control (A,C) and Chd4fl/fl;Tie2-Cre+ (B,D) hearts were stained with Alcian blue to detect acidic glycosaminoglycans that contribute to cardiac ECM. Arrow in (B) indicates an area of decreased Alcian blue staining in a mutant ventricle. Arrow in (D) indicates a hypotrabeculated region of a mutant ventricle that also has diminished Alcian blue staining. Images are representative of results from comparisons between 3 littermate control and mutant embryos. Scale bars: 100 µm. (TIF) [file pgen.1004031.s003.tif]

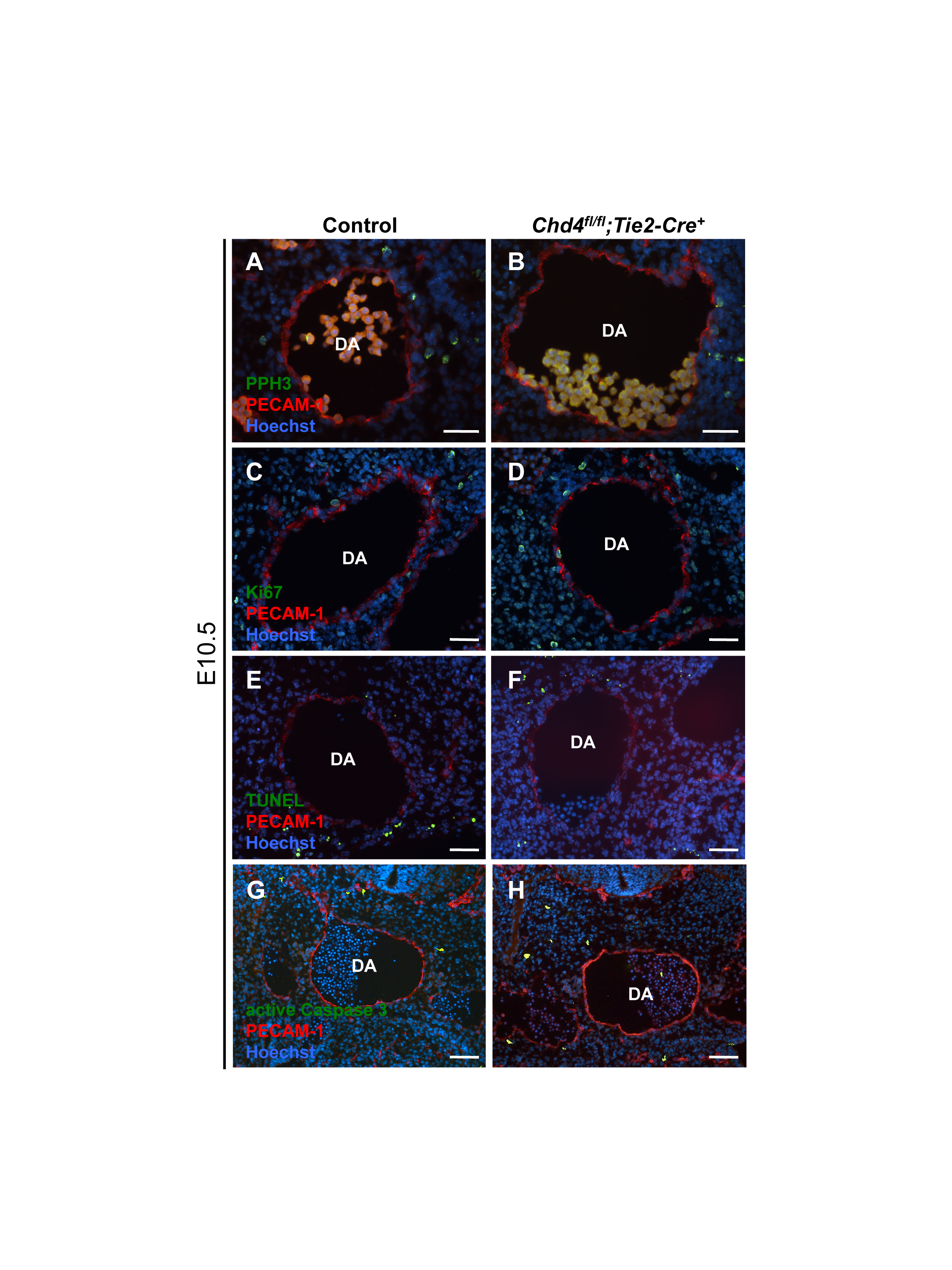

Supplement: Figure S4 — Endothelial cell proliferation and apoptosis are normal in Chd4fl/fl;Tie2-Cre+ rupture-prone vessels. (A–H) Histological sections of dorsal aortae (DA) from E10.5 littermate control (A,C,E,G) and Chd4fl/fl;Tie2-Cre+ (B,D,F,H) embryos were stained to assess endothelial cell proliferation or apoptosis. Immunostaining with anti-PECAM-1 antibodies (red) was used to visualize the vasculature and Hoechst dye (blue) was used to stain nuclei. Proliferation was evaluated by immunostaining with anti-phosphorylated histone H3 (PPH3; A,B) or anti-Ki67 (C,D) antibodies. Apoptosis was detected by TUNEL staining (E,F) or by immunostaining with anti-active caspase 3 antibodies (G,H). Representative images from three separate experiments are shown; no quantitative differences were detected in staining for PPH3 (0/116 control vs. 0/126 mutant endothelial cells), Ki67 (0/54 control vs. 1/51 mutant endothelial cells), TUNEL (0/136 control vs. 0/121 mutant endothelial cells), or active caspase 3 (1/151 vs. 0/126 endothelial cells). Scale bars: 100 µm. (TIF) [file pgen.1004031.s004.tif]

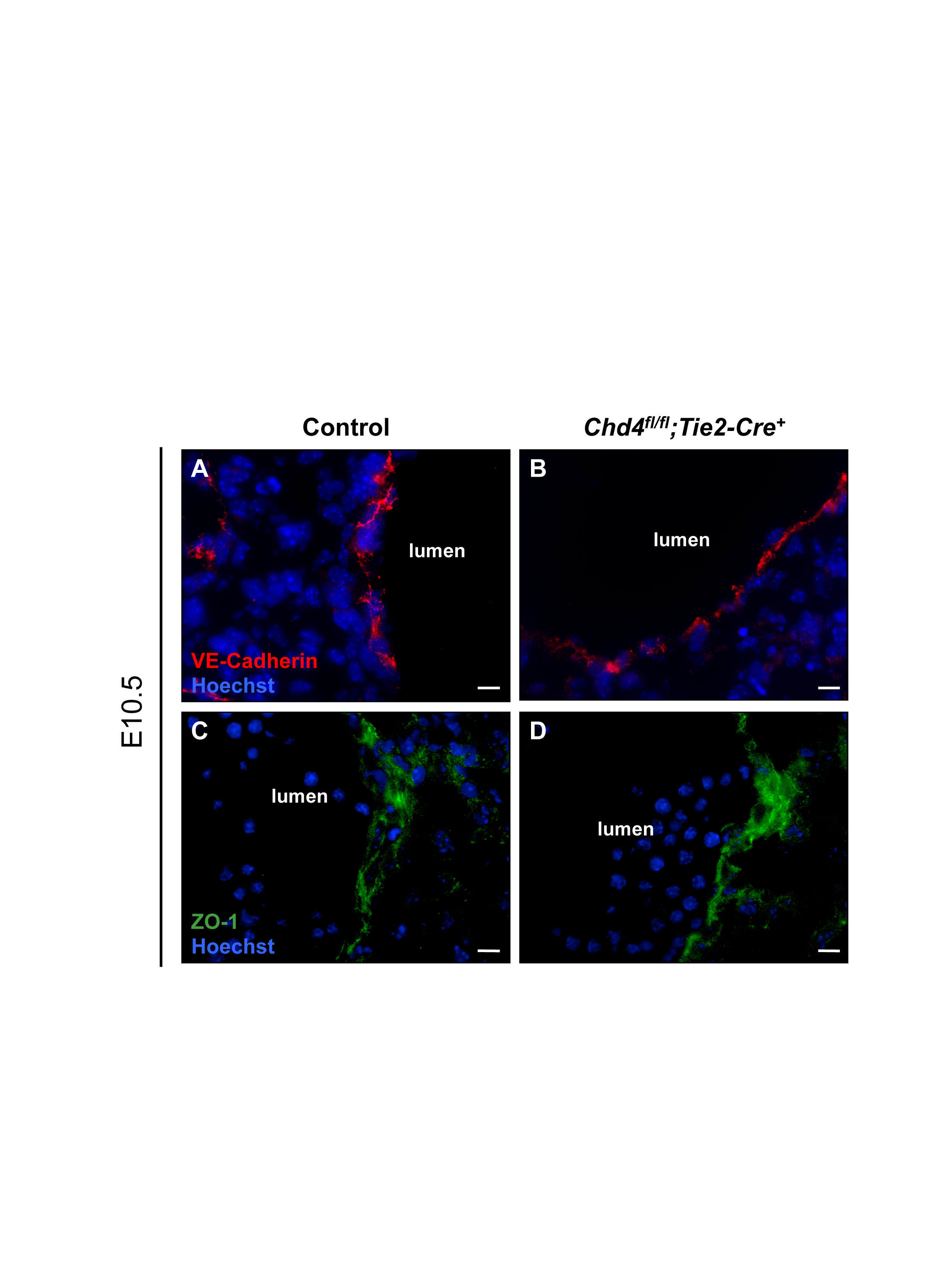

Supplement: Figure S5 — Control and Chd4fl/fl;Tie2-Cre+ endothelial cells lining rupture-prone vessels express comparable levels of intercellular junction markers at E10.5. (A–D) Cryosections of dorsal aortae from E10.5 littermate control (A,C) and Chd4fl/fl;Tie2-Cre+ (B,D) embryos were immunostained for the adherens junction marker VE-Cadherin (red; A,B) or the tight junction marker ZO-1 (green; C,D) and analyzed by confocal microscopy. Hoechst (blue) was used as a nuclear counterstain. Representative images from three independent experiments are shown. Scale bars: 10 µm. (TIF) [file pgen.1004031.s005.tif]

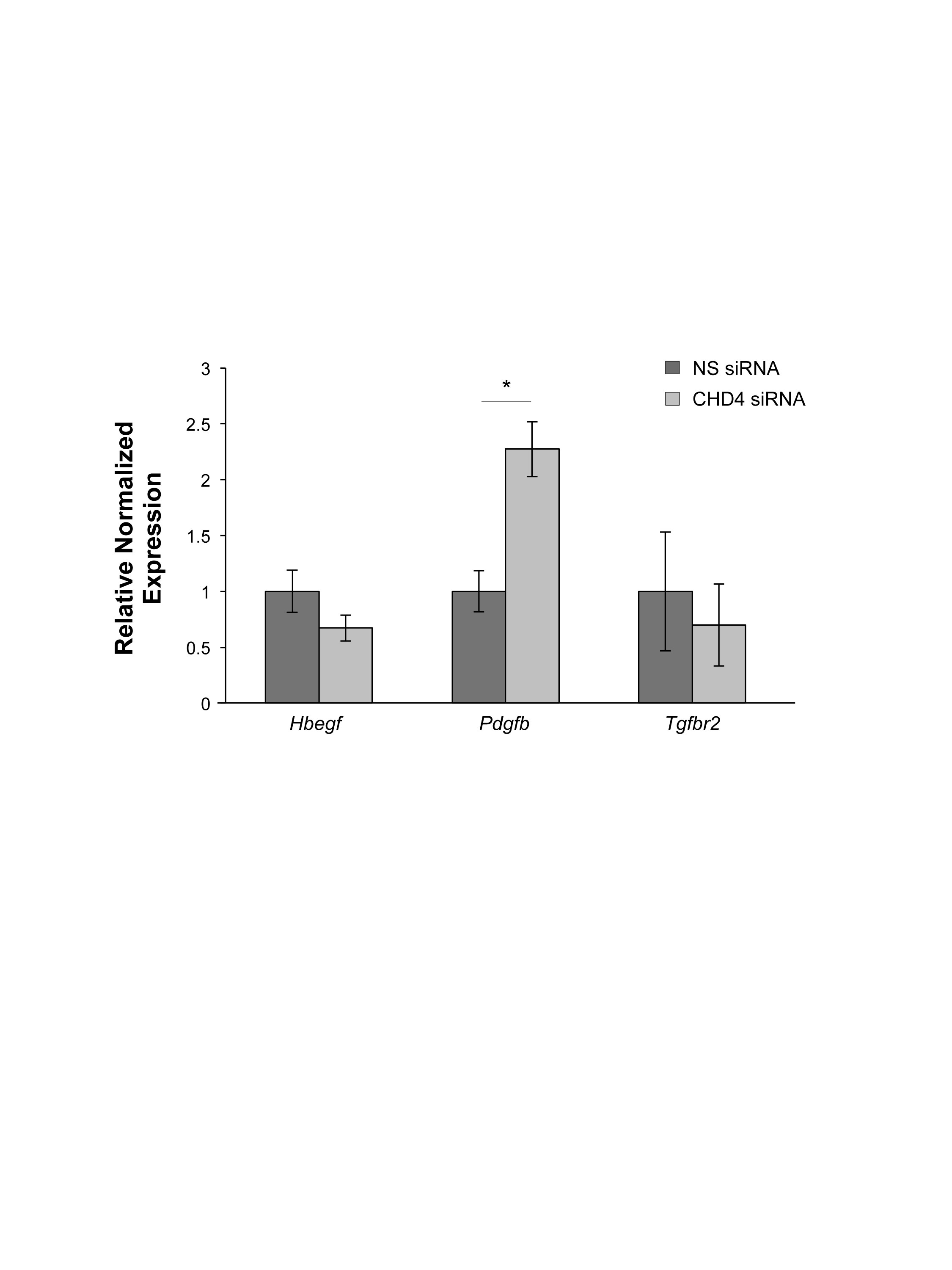

Supplement: Figure S6 — Genes involved in smooth muscle cell recruitment toward endothelium are expressed at normal or elevated levels in CHD4 knockdown endothelial cells. C166 endothelial cells were transfected with nonspecific (NS) or CHD4-specific siRNA oligonucleotides for 24 h. RNA was isolated, cDNA was synthesized, and qPCR was performed using gene-specific primers for heparin-binding EGF-like growth factor (Hbegf), platelet derived growth factor B (Pdgfb), or transforming growth factor β receptor II (Tgfbr2). Data were normalized to the relative expression of NS siRNA-treated samples. Error bars represent ± SD of results from three independent experiments. Statistical analysis was performed using a two-tailed Student's t test (*, p<0.05). (TIF) [file pgen.1004031.s006.tif]

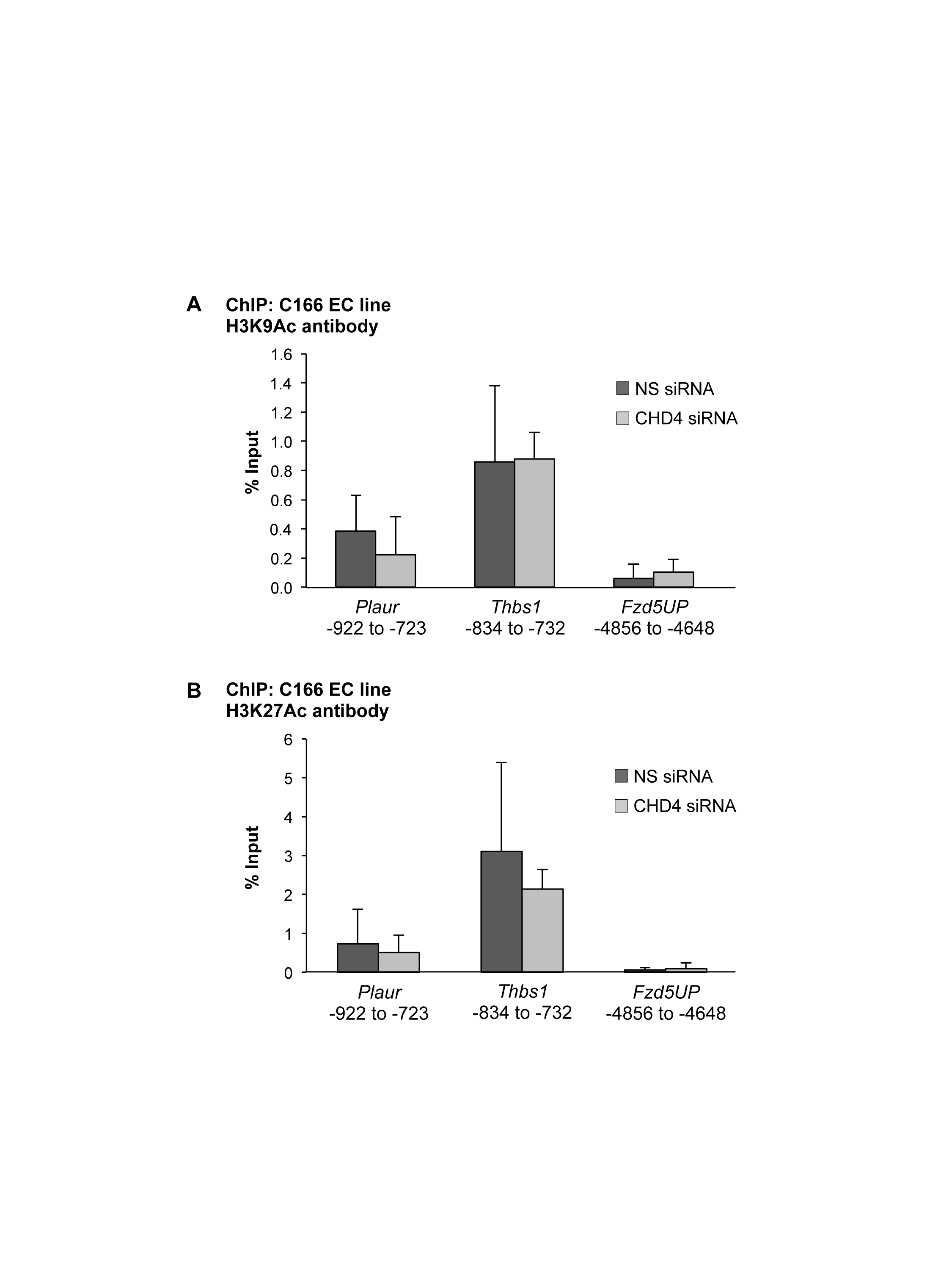

Supplement: Figure S7 — No changes in H3K9Ac or H3K27Ac enrichment are seen at the Plaur and Thbs1 promoters in CHD4 knockdown endothelial cells. Chromatin immunoprecipitation (ChIP) assays were carried out in C166 endothelial cells transfected for 24 h with either non-specific (NS) siRNA or CHD4-specific siRNA oligonucleotides. Immunoprecipitation was performed using antibodies against H3K9Ac (A) or H3K27Ac (B). Immunoprecipitated DNA was analyzed by qPCR to examine enrichment of H3K9Ac or H3K27Ac at the Plaur and Thbs1 promoters. A transcriptionally inactive region approximately 5 kb upstream of the Fzd5 transcription start site (Fzd5UP) was assessed as a negative control for antibody binding. Data are represented as a percent of total input chromatin. Error bars represent SD of results from three independent experiments. For statistical analysis, NS siRNA- and CHD4 siRNA-treated samples were compared at each locus using a two-tailed Student's t test; no significant differences were detected. Likewise, no significant differences were detected between NS siRNA- and CHD4 siRNA-treated samples when H3K9Ac and H3K27Ac marks were normalized against total H3 pulldown at the Plaur and Thbs1 promoters (data not shown). (TIF) [file pgen.1004031.s007.tif]

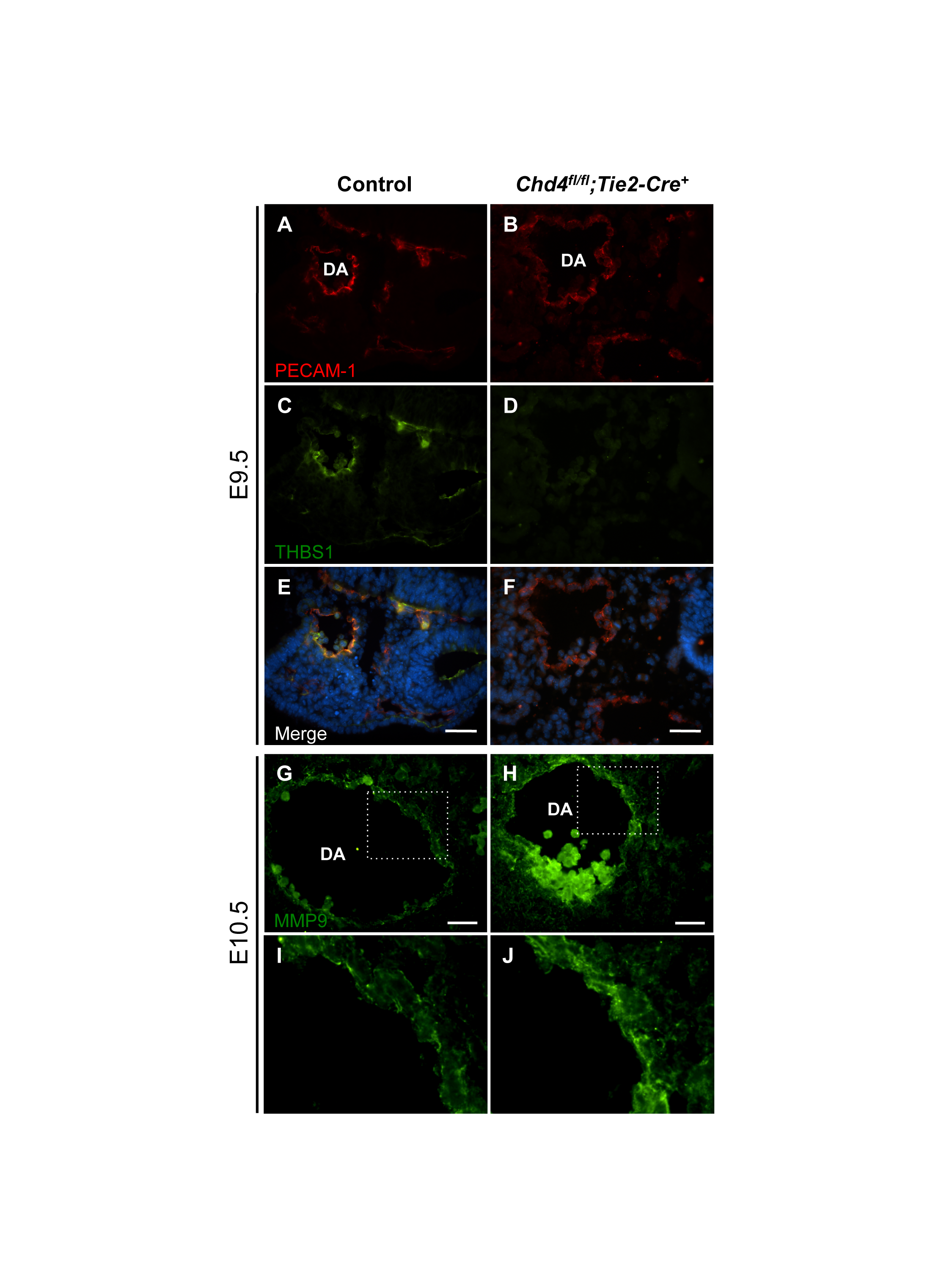

Supplement: Figure S8 — Thrombospondin and its downstream target MMP9 are misexpressed around rupture-prone Chd4fl/fl;Tie2-Cre+ dorsal aortae. (A–F) Cryosections of dorsal aortae (DA) from E9.5 littermate control (A,C,E) and Chd4fl/fl;Tie2-Cre+ (B,D,F) embryos were stained with anti-PECAM-1 antibodies (red; A,B), anti-THBS1 antibodies (green; C,D), and Hoechst (blue; E,F). Merged images are shown in panels E and F. (G–J) Cryosections of dorsal aortae from E10.5 littermate control (G, I) and Chd4fl/fl;Tie2-Cre+ (H,J) embryos were stained with anti-MMP9 antibodies. The boxed regions in panels G and H are magnified in panels I and J, respectively. Scale bars: 50 µm. (TIF) [file pgen.1004031.s008.tif]

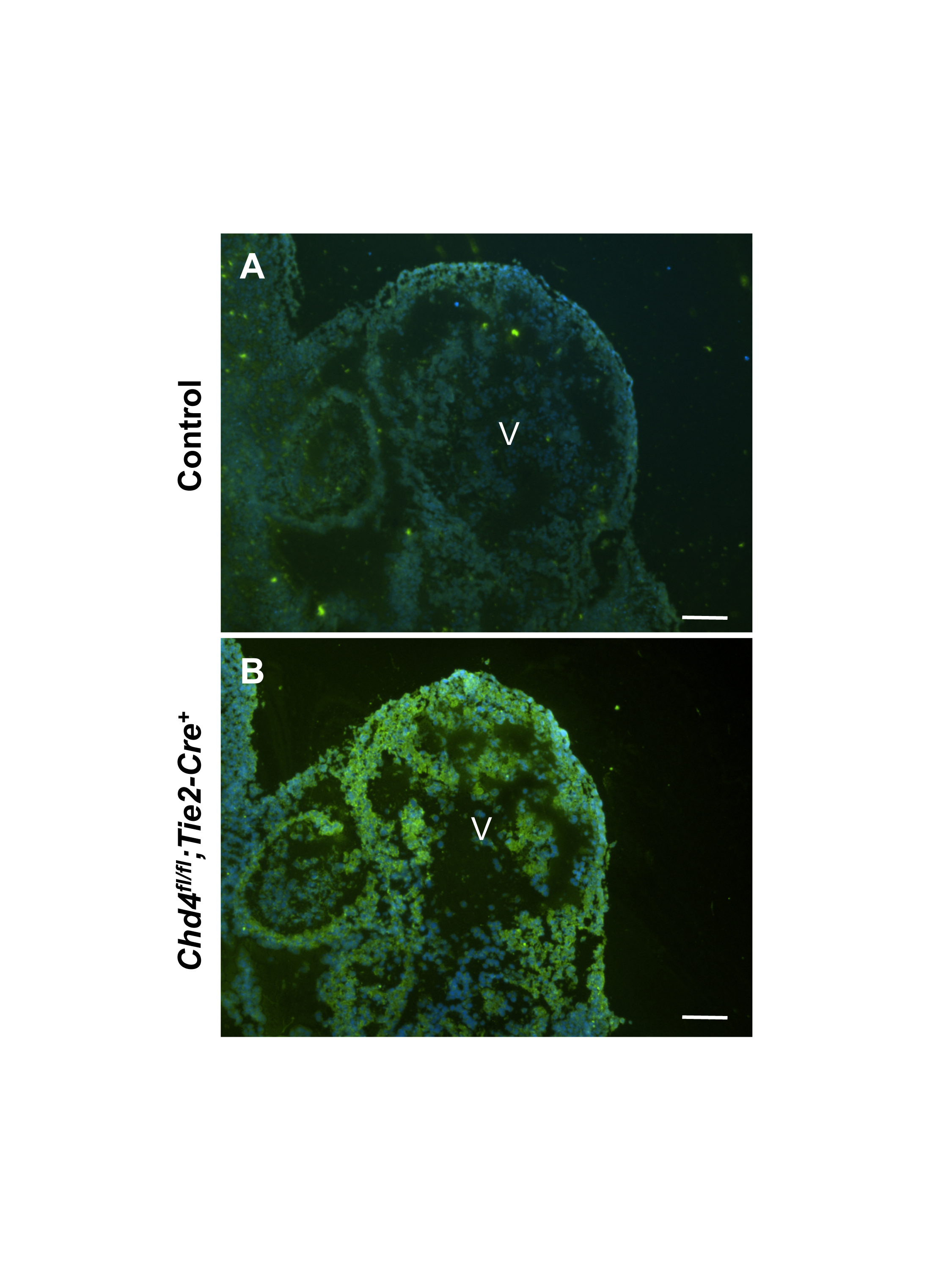

Supplement: Figure S9 — Plasmin activity is elevated in E10.5 Chd4fl/fl;Tie2-Cre+ hearts. In situ zymography was performed on sections of E10.5 littermate control (A) and Chd4fl/fl;Tie2-Cre+ (B) embryonic hearts for detection of plasmin activity, as described in Figure 5. Casein cleavage (green fluorescence) was substantially higher in the Chd4fl/fl;Tie2-Cre+ hearts versus the control hearts. Hoechst (blue) was used as a nuclear counterstain. V = ventricle. Scale bars: 100 µm. (TIF) [file pgen.1004031.s009.tif]

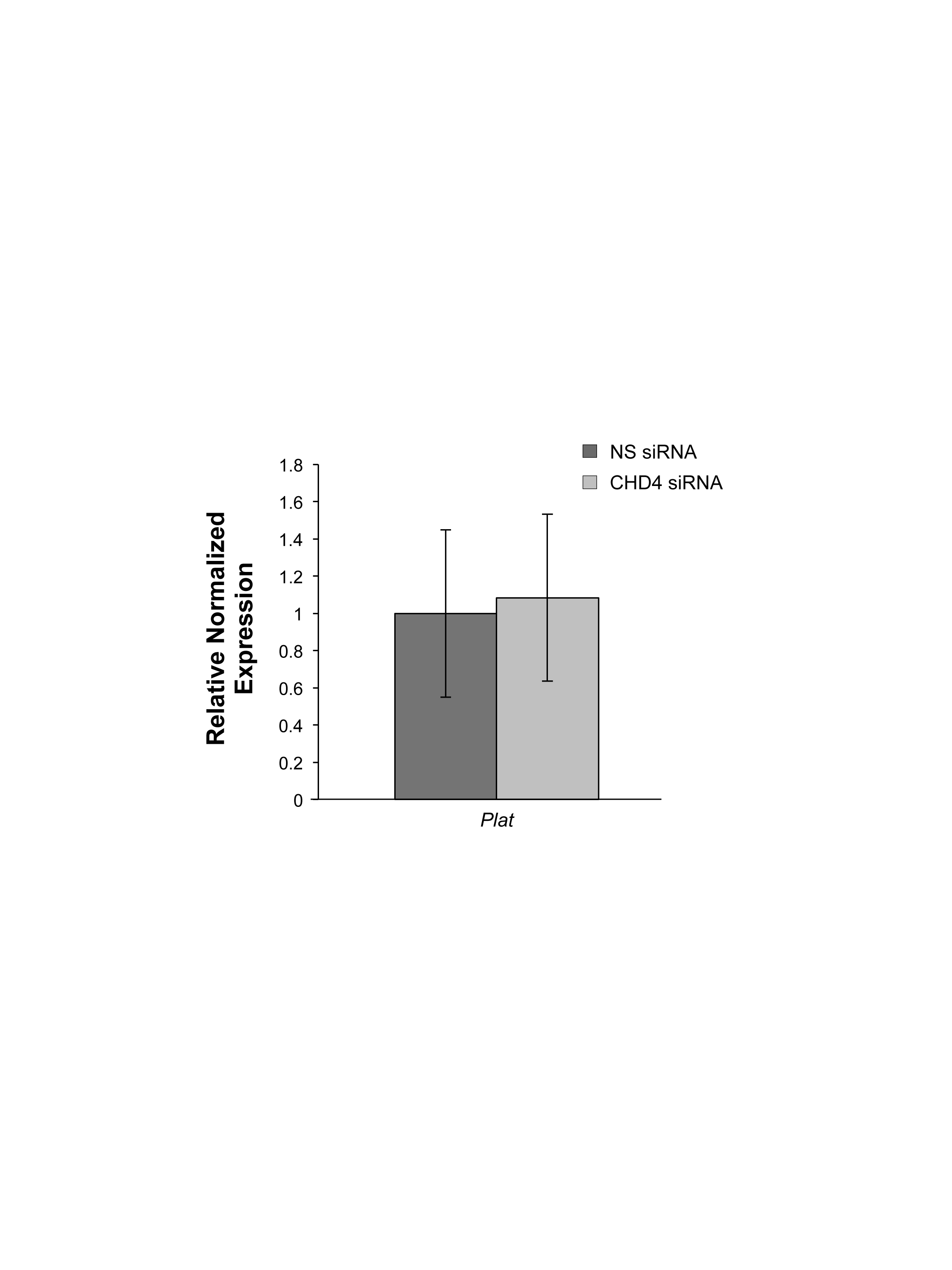

Supplement: Figure S10 — Expression of tissue plasminogen activator (tPA; Plat) is normal in CHD4 knockdown endothelial cells. C166 endothelial cells were transfected with nonspecific (NS) or CHD4-specific siRNA oligonucleotides for 24 h. RNA was isolated, cDNA was synthesized, and qPCR was performed using Plat-specific primers. Data were normalized to the relative expression of NS siRNA-treated samples. Error bars represent ± SD of results from three independent experiments. (TIF) [file pgen.1004031.s010.tif]

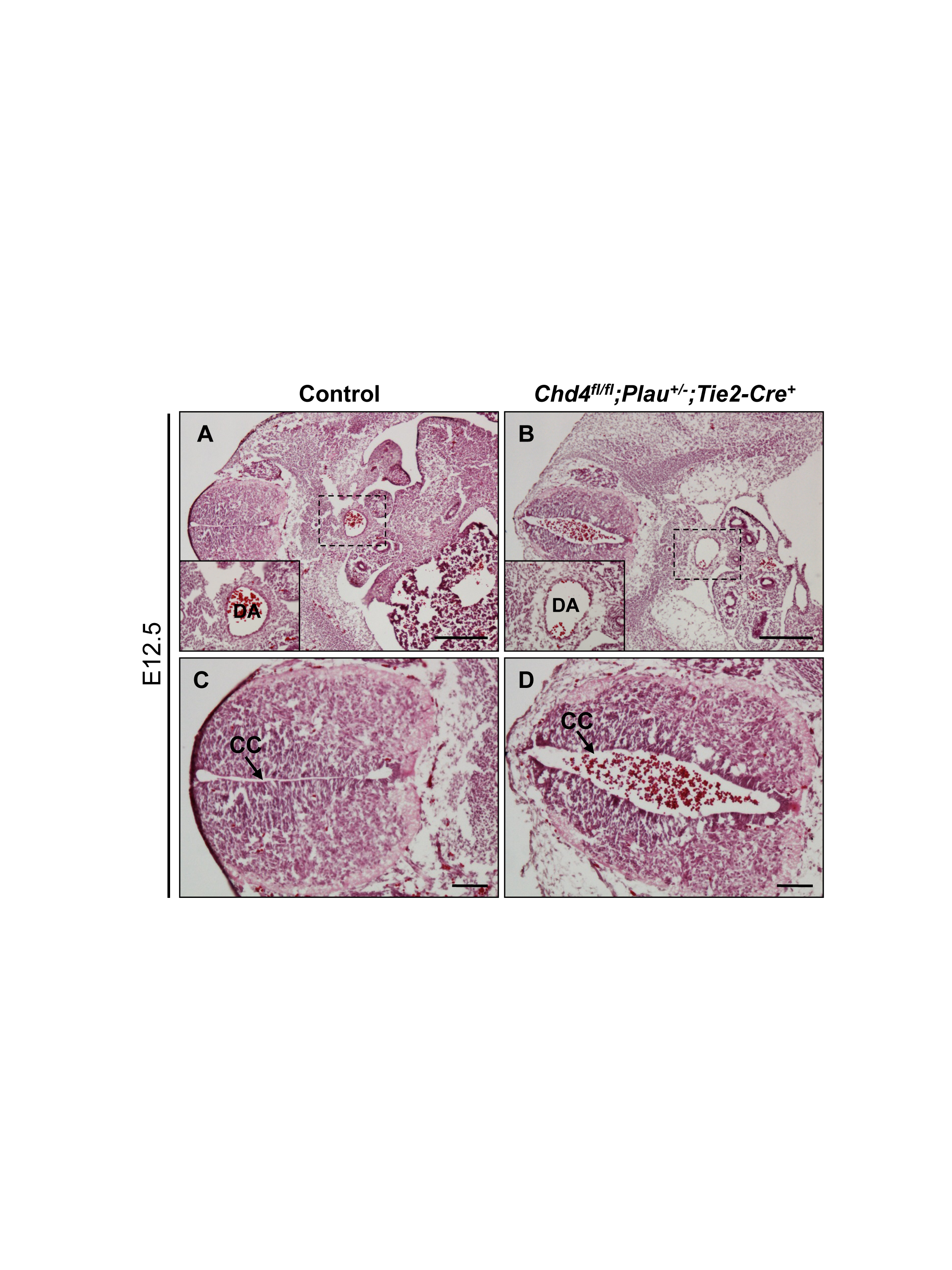

Supplement: Figure S11 — Rupture-prone dorsal aortae are significantly rescued from hemorrhage in Chd4fl/fl;Plau+/−;Tie2-Cre+ embryos. (A–D) Hematoxylin and eosin (H&E) staining of E12.5 littermate control and Chd4fl/fl;Plau+/−;Tie2-Cre+ embryos revealed an intact dorsal aorta (DA; insets in panels A and B). However, blood was seen aberrantly pooling in the central canal (CC) of the spinal cord in Chd4fl/fl;Plau+/−;Tie2-Cre+ embryos (D). Scale bars: 500 µm (A–B); 100 µm (C–D). (TIF) [file pgen.1004031.s011.tif]
