# Supplementary material for: The NuRD Chromatin-Remodeling Enzyme CHD4 Promotes Embryonic Vascular Integrity by Transcriptionally Regulating Extracellular Matrix Proteolysis
Source: PLoS Genet. 2013 Dec 12;9(12):e1004031. doi: 10.1371/journal.pgen.1004031 (PMC3861115; doi:10.1371/journal.pgen.1004031)
Supplement: Table S1 — Genes with unchanged expression levels in E10.5 Chd4fl/fl;Tie2-Cre+ endothelial cells. Endothelial cells from E10.5 littermate control and Chd4fl/fl;Tie2-Cre+ embryos were isolated, RNA was purified, cDNA was synthesized and qPCR was performed using two commercial qPCR arrays containing a total of 157 genes important for extracellular matrix composition and angiogenesis (SABiosciences/QIAGEN). The 148 genes that were identified as having insignificant changes in expression levels (p>0.1) after three different experiments are listed. Data analysis was performed using the web-based PCR Array Data Analysis tool recommended for use with these arrays: (http://www.sabiosciences.com/pcrarraydataanalysis.php). See Table S2 for the list of genes with significant expression changes from the arrays. (DOC) [file pgen.1004031.s012.doc]

**Genes with unchanged expression levels in E10.5 *Chd4^fl/fl^;Tie2-Cre^+^* endothelial cells**

| **Gene**  **Symbol** | **Description** |
| --- | --- |
| *Adamts1* | A disintegrin-like and metallopeptidase with thrombospondin type 1 motif, 1 |
| *Adamts2* | A disintegrin-like and metallopeptidase with thrombospondin type 1 motif, 2 |
| *Adamts5* | A disintegrin-like and metallopeptidase with thrombospondin type 1 motif, 5 |
| *Adamts8* | A disintegrin-like and metallopeptidase with thrombospondin type 1 motif, 8 |
| *Angpt1* | Angiopoietin 1 |
| *Angpt2* | Angiopoietin 2 |
| *Anpep* | Alanyl (membrane) aminopeptidase |
| *Bai1* | Brain-specific angiogenesis inhibitor 1 |
| *Ccl11* | Chemokine (C-C motif) ligand 11 |
| *Ccl2* | Chemokine (C-C motif) ligand 2 |
| *Cd44* | CD44 antigen |
| *Cdh1* | Cadherin 1 |
| *Cdh2* | Cadherin 2 |
| *Cdh3* | Cadherin 3 |
| *Cdh4* | Cadherin 4 |
| *Cdh5* | Cadherin 5 (VE-Cadherin) |
| *Cntn1* | Contactin 1 |
| *Col181* | Collagen, type XVIII, alpha 1 |
| *Col1a1* | Collagen, type I, alpha 1 |
| *Col2a1* | Collagen, type II, alpha 1 |
| *Col3a1* | Collagen, type III, alpha 1 |
| *Col4a1* | Collagen, type IV, alpha 1 |
| *Col4a2* | Collagen, type IV, alpha 2 |
| *Col4a3* | Collagen, type IV, alpha 3 |
| *Col5a1* | Collagen, type V, alpha 1 |
| *Col6a1* | Collagen, type VI, alpha 1 |
| *Csf3* | Colony stimulating factor 3 (granulocyte) |
| *Ctgf* | Connective tissue growth factor |
| *Ctnna1* | Catenin (cadherin associated protein), alpha 1 |
| *Ctnna2* | Catenin (cadherin associated protein), alpha 2 |
| *Ctnnb1* | Catenin (cadherin associated protein), beta 1 |
| *Cxcl1* | Chemokine (C-X-C motif) ligand 1 |
| *Cxcl2* | Chemokine (C-X-C motif) ligand 2 |
| *Cxcl5* | Chemokine (C-X-C motif) ligand 5 |
| *Ecm1* | Extracellular matrix protein 1 |
| *Efna1* | Ephrin A1 |
| *Efnb2* | Ephrin B2 |
| *Egf* | Epidermal growth factor |
| *Emilin1* | Elastin microfibril interfacer 1 |
| *Eng* | Endoglin |
| *Entpd1* | Ectonucleoside triphosphate diphosphohydrolase 1 |
| *Epas1* | Endothelial PAS domain protein 1 |
| *Ephb4* | Eph receptor B4 |
| *Ereg* | Epiregulin |
| *F2* | Coagulation factor II (prothrombin) |
| *Fbln1* | Fibulin 1 |
| *Fgf1* | Fibroblast growth factor 1 |
| *Fgf2* | Fibroblast growth factor 2 |
| *Fgf6* | Fibroblast growth factor 6 |
| *Fgfr3* | Fibroblast growth factor receptor 3 |
| *Figf* | C-fos induced growth factor |
| *Flt1* | FMS-like tyrosine kinase 1 |
| *Fn1* | Fibronectin 1 |
| *Fzd5* | Frizzled homolog 5 (Drosophila) |
| *Gna13* | Guanine nucleotide binding protein, alpha 13 |
| *Hand2* | Heart and neural crest derivatives expressed transcript 2 |
| *Hapln1* | Hyaluronan and proteoglycan link protein 1 |
| *Hc* | Hemolytic complement |
| *Hgf* | Hepatocyte growth factor |
| *Hif1a* | Hypoxia inducible factor 1, alpha subunit |
| *Icam1* | Intercellular adhesion molecule 1 |
| *Ifng* | Interferon gamma |
| *Igf1* | Insulin-like growth factor 1 |
| *Il1b* | Interleukin 1 beta |
| *Il6* | Interleukin 6 |
| *Itga2* | Integrin alpha 2 |
| *Itga3* | Integrin alpha 3 |
| *Itga4* | Integrin alpha 4 |
| *Itga5* | Integrin alpha 5 (fibronectin receptor alpha) |
| *Itgae* | Integrin alpha E, epithelial-associated |
| *Itgal* | Integrin alpha L |
| *Itgam* | Integrin alpha M |
| *Itgav* | Integrin alpha V |
| *Itgax* | Integrin alpha X |
| *Itgb1* | Integrin beta 1 (fibronectin receptor beta) |
| *Itgb2* | Integrin beta 2 |
| *Itgb4* | Integrin beta 4 |
| *Jag1* | Jagged 1 |
| *Kdr* | Kinase insert domain protein receptor (VEGFR2) |
| *Lama1* | Laminin, alpha 1 |
| *Lama2* | Laminin, alpha 2 |
| *Lama3* | Laminin, alpha 3 |
| *Lama5* | Laminin, alpha 5 |
| *Lamb2* | Laminin, beta 2 |
| *Lamb3* | Laminin, beta 3 |
| *Lamc1* | Laminin, gamma 1 |
| *Lect1* | Leukocyte cell derived chemotaxin 1 |
| *Lep* | Leptin |
| *Mapk14* | Mitogen-activated protein kinase 14 |
| *Mmp10* | Matrix metallopeptidase 10 |
| *Mmp11* | Matrix metallopeptidase 11 |
| *Mmp12* | Matrix metallopeptidase 12 |
| *Mmp13* | Matrix metallopeptidase 13 |
| *Mmp14* | Matrix metallopeptidase 14 (membrane-inserted) |
| *Mmp15* | Matrix metallopeptidase 15 |
| *Mmp19* | Matrix metallopeptidase 19 |
| *Mmp1a* | Matrix metallopeptidase 1a (interstitial collagenase) |
| *Mmp3* | Matrix metallopeptidase 3 |
| *Mmp7* | Matrix metallopeptidase 7 |
| *Mmp8* | Matrix metallopeptidase 8 |
| *Mmp9* | Matrix metallopeptidase 9 |
| *Ncam1* | Neural cell adhesion molecule 1 |
| *Ncam2* | Neural cell adhesion molecule 2 |
| *Npr1* | Natriuretic peptide receptor 1 |
| *Nrp1* | Neuropilin 1 |
| *Nrp2* | Neuropilin 2 |
| *Pdgfa* | Platelet derived growth factor, alpha |
| *Pecam1* | Platelet/endothelial cell adhesion molecule 1 |
| *Pgf* | Placental growth factor |
| *Plg* | Plasminogen |
| *Plxdc1* | Plexin domain containing 1 |
| *Postn* | Periostin, osteoblast specific factor |
| *Ptgs1* | Prostaglandin-endoperoxide synthase 1 |
| *S1pr1* | Sphingosine-1-phosphate receptor 1 |
| *Sele* | Selectin, endothelial cell |
| *Sell* | Selectin, lymphocyte |
| *Selp* | Selectin, platelet |
| *Serpinf1* | Serine peptidase inhibitor, clade F, member 1 (Pedf) |
| *Sgce* | Sarcoglycan, epsilon |
| *Sparc* | Secreted acidic cysteine rich glycoprotein |
| *Sphk1* | Sphingosine kinase 1 |
| *Spock1* | Sparc/osteonectin, cwcv and kazal-like domains proteoglycan 1 (Testican 1) |
| *Spp1* | Secreted phosphoprotein 1 |
| *Stab1* | Stabilin 1 |
| *Syt1* | Synaptotagmin I |
| *Tbx4* | T-box 4 |
| *Tgfa* | Transforming growth factor alpha |
| *Tgfb1* | Transforming growth factor, beta 1 |
| *Tgfb2* | Transforming growth factor, beta 2 |
| *Tgfb3* | Transforming growth factor, beta 3 |
| *Tgfbi* | Transforming growth factor, beta induced |
| *Tgfbr1* | Transforming growth factor, beta receptor I |
| *Thbs2* | Thrombospondin 2 |
| *Thbs3* | Thrombospondin 3 |
| *Timp1* | Tissue inhibitor of metalloproteinase 1 |
| *Timp2* | Tissue inhibitor of metalloproteinase 2 |
| *Timp3* | Tissue inhibitor of metalloproteinase 3 |
| *Tmprss6* | Transmembrane serine protease 6 |
| *Tnc* | Tenascin C |
| *Tnf* | Tumor necrosis factor |
| *Tnfaip2* | Tumor necrosis factor, alpha-induced protein 2 |
| *Tnfsf12* | Tumor necrosis factor (ligand) superfamily, member 12 |
| *Tymp* | Thymidine phosphorylase |
| *Vcam1* | Vascular cell adhesion molecule 1 |
| *Vcan* | Versican |
| *Vegfa* | Vascular endothelial growth factor A |
| *Vegfb* | Vascular endothelial growth factor B |
| *Vtn* | Vitronectin |
