# Supplementary material for: The NuRD Chromatin-Remodeling Enzyme CHD4 Promotes Embryonic Vascular Integrity by Transcriptionally Regulating Extracellular Matrix Proteolysis
Source: PLoS Genet. 2013 Dec 12;9(12):e1004031. doi: 10.1371/journal.pgen.1004031 (PMC3861115; doi:10.1371/journal.pgen.1004031)
Supplement: Table S2 — Genes misregulated in E10.5 Chd4fl/fl;Tie2-Cre+ embryonic endothelial cells. Endothelial cells from E10.5 littermate control and Chd4fl/fl;Tie2-Cre+ embryos were isolated, RNA was purified, cDNA was synthesized and qPCR was performed using two commercial qPCR arrays containing a total of 157 genes important for extracellular matrix composition and angiogenesis (SABiosciences/QIAGEN). The nine genes that were identified as having significantly differential expression levels (p<0.1) after three different experiments are listed along with their average fold change. Data analysis was performed using the web-based PCR Array Data Analysis tool recommended for use with these arrays: (http://www.sabiosciences.com/pcrarraydataanalysis.php). See Table S1 for the list of genes with insignificant expression changes from the arrays. (DOC) [file pgen.1004031.s013.doc]

**Genes misregulated in E10.5 *Chd4^fl/fl^;Tie2-Cre^+^* embryonic endothelial cells**

**p*<0.1

| **Fold Change*** | **Gene**  **Symbol** | **Description** |
| --- | --- | --- |
| 2.1 | *Plau* | Plasminogen activator, urokinase |
| 1.5 | *Vegfc* | Vascular endothelial growth factor C |
| -1.2 | *Mmp2* | Matrix metallopeptidase 2 |
| -1.2 | *Tbx1* | T-box 1 |
| -1.3 | *Mdk* | Midkine |
| -1.4 | *Tek* | Endothelial-specific receptor tyrosine kinase (Tie2) |
| -1.9 | *Smad5* | MAD homolog 5 |
| -4.1 | *Thbs1* | Thrombospondin 1 |
| -7.7 | *Itgb3* | Integrin beta 3 |
